# Modeling of the Concentrations of Ultrafine Particles in the Plumes of Ships in the Vicinity of Major Harbors

**DOI:** 10.3390/ijerph17030777

**Published:** 2020-01-26

**Authors:** Matthias Karl, Liisa Pirjola, Ari Karppinen, Jukka-Pekka Jalkanen, Martin Otto Paul Ramacher, Jaakko Kukkonen

**Affiliations:** 1 Chemistry Transport Modelling, Helmholtz Zentrum Geesthacht, 21502 Geesthacht, Germany; martin.ramacher@hzg.de; 2 Department of Technology, Metropolia University of Applied Sciences, P.O. Box 4071, FI-01600 Vantaa, Finland; liisa.pirjola@metropolia.fi; 3 Department of Physics, University of Helsinki, P.O. Box 64, 00014 Helsinki, Finland; 4 Atmospheric Composition Research, Finnish Meteorological Institute, P.O. Box 503, 00101 Helsinki, Finland; ari.karppinen@fmi.fi (A.K.); jukka-pekka.jalkanen@fmi.fi (J.-P.J.); jaakko.kukkonen@fmi.fi (J.K.)

**Keywords:** shipping emissions, ultrafine particles, fuel sulfur content, aerosol dynamics modeling, atmospheric nucleation, particle number concentration, air quality, population exposure

## Abstract

Marine traffic in harbors can be responsible for significant atmospheric concentrations of ultrafine particles (UFPs), which have widely recognized negative effects on human health. It is therefore essential to model and measure the time evolution of the number size distributions and chemical composition of UFPs in ship exhaust to assess the resulting exposure in the vicinity of shipping routes. In this study, a sequential modelling chain was developed and applied, in combination with the data measured and collected in major harbor areas in the cities of Helsinki and Turku in Finland, during winter and summer in 2010–2011. The models described ship emissions, atmospheric dispersion, and aerosol dynamics, complemented with a time–microenvironment–activity model to estimate the short-term UFP exposure. We estimated the dilution ratio during the initial fast expansion of the exhaust plume to be approximately equal to eight. This dispersion regime resulted in a fully formed nucleation mode (denoted as Nuc_2_). Different selected modelling assumptions about the chemical composition of Nuc_2_ did not have an effect on the formation of nucleation mode particles. Aerosol model simulations of the dispersing ship plume also revealed a partially formed nucleation mode (Nuc_1_; peaking at 1.5 nm), consisting of freshly nucleated sulfate particles and condensed organics that were produced within the first few seconds. However, subsequent growth of the new particles was limited, due to efficient scavenging by the larger particles originating from the ship exhaust. The transport of UFPs downwind of the ship track increased the hourly mean UFP concentrations in the neighboring residential areas by a factor of two or more up to a distance of 3600 m, compared with the corresponding UFP concentrations in the urban background. The substantially increased UFP concentrations due to ship traffic significantly affected the daily mean exposures in residential areas located in the vicinity of the harbors.

## 1. Introduction

Large ships are primarily powered by diesel propulsion systems fueled by heavy fuel oil (HFO) [1]. HFO is la ow-grade fuel, also known as bunker oil or residual oil, which usually has a fuel sulfur content (FSC) of over 0.5% and includes a high concentration of impurities, such as ash, asphaltenes, and metals [2,3]. Sulphur oxides (SO_X_) emitted from ship engines form sulfate (SO_4_) aerosols in the atmosphere that increase human health risks and contribute to the acidification in terrestrial and aquatic environments [4]. It has been estimated that ship emissions contribute between 5% and 20% of ambient non-sea-salt sulfate concentrations and 5% to 30% of sulfur dioxide (SO_2_) concentrations in coastal regions [5]. SO_4_ constitutes a large proportion of ship-related particulate matter (PM). The most important health effect associated with PM exposure is premature mortality [6]. Specifically, increases in concentrations of PM_2.5_, i.e., particles with aerodynamic diameter of 2.5 μm (2.5 × 10^−6^ m) or less, have been linked to increases in cardiopulmonary and lung cancer mortalities in exposed populations [7]. PM emissions from ship traffic are responsible for approximately 60,000 premature deaths globally [8]. According to Sofiev et al. [9], the worldwide use of cleaner marine fuels with a lower content of sulfur will strongly reduce the ship-related premature mortality and morbidity by 34% and 54%, respectively.

High concentrations of ultrafine particles (UFPs), defined as particles having mobility diameter (Dp) less than 100 nm (1.0 × 10^−7^ m), have been measured in marine ports in Europe [10,11,12,13]. Due to their small size, UFP can penetrate deeper in the respiratory tract than larger particles. Physical characteristics related to UFP size, such as a high deposition efficiency and high active surface, together with their chemical composition imply that the health effects of UFP might be independent from those of larger particles, such as PM_2.5_ [14,15]. UFP are not well recognized by the human immune system, cleared less efficiently in the lung than larger particles, have high biological reactivity, and can reach the bloodstream [14,16]. By crossing through the alveolar–blood barrier, UFP may interact with the vascular endothelium, causing local oxidative stress with resultant acute cardiovascular events [17]. The blood circulation translocates ultrafine particles from the lungs to other critical organs, such as the heart, liver, kidneys, and brain, where they can induce localized effects [18]. However, the few epidemiological studies that have been carried out to investigate the effect of UFP on mortality revealed inconsistent results, one reason being that the current exposure assessment techniques are not well suited to describe the long-term exposure to UFPs [19].

Modeling of regional and urban air quality showed that the influence of shipping and harbors was relevant on particle number concentrations for Helsinki, Oslo, Rotterdam, and Athens [20]. Ship engine exhaust aerosol is composed of ultrafine combustion particles consisting of soot and volatile material [21,22]. Due to different particle formation mechanisms and characteristics, the number size distributions of fresh exhaust particles from marine diesel engines consist of distinctive particle modes. Particle size number distributions from ship emissions are defined by a nucleation mode of particles smaller than 25 nm and an accumulation mode of particles in the size range 25−1000 nm, with only a minor fraction of particles above 250 nm [21,23,24,25]. Although the boundary between the two modes can vary [26], we adopted this definition of the nucleation mode in our study. Moldanová et al. [27] measured the emission factors of particle numbers from a four-stroke engine fueled with HFO and marine gas oil (MGO) in the range 5 × 10^15^–1 × 10^17^ (kg fuel)^−1^, depending on the engine load. The number concentration was dominated by UFPs, and ca. 2/3 of the particles were non-volatile. Nucleation mode particles in the ship exhaust are presumably volatile and composed of sulfuric acid water clusters [21].

This study focuses on nucleation mode particles that can be emitted either (i) directly from the ship’s engine or (ii) formed via atmospheric nucleation in the ship’s plume. During combustion, fuel sulfur is oxidized to SO_2_ and further to sulfur trioxide, SO_3_ [12]. The amount of SO_3_ depends on the combustion temperature. The oxidation of SO_2_ to SO_3_ in the cylinder of the ship engine is dominated by gas phase reactions while the vanadium catalyzed reaction is a very minor pathway [28]. After the release at the exit of the ship stack, in the dilution and cooling of the exhaust, SO_3_ reacts with water vapor, forming sulfuric acid (H_2_SO_4_), which can subsequently homogeneously nucleate to form new particles and/or condense onto pre-existing particles. As a consequence, SO_X_ emitted from ships can be involved in new particle formation (NPF) in coastal cities [29]. Ship exhaust also contains low volatile organic compounds that can contribute both to the nucleation processes and to the subsequent particle growth [30]. NFP events occur frequently in coastal areas because the mixing of clean marine air and polluted urban air gives rise to favorable conditions for particle formation, often characterized by high solar radiation, and periods of land–sea breeze transitions [31].

A previous study by Tian et al. [32] presented a model system that simulated the transformation of fresh ship emissions during atmospheric ageing of plumes for several hours. The applied stochastic Lagrangian particle-resolved aerosol model was able to capture the aerosol dynamics during plume evolution. However, they did not consider the fast interactions between particle dynamics and dilution on the shorter time scales in the early phase of the plume dispersion. In addition, their model yielded higher particle number concentrations below the diameter of 40 nm, compared to aircraft observations of ship plumes that had aged for one hour.

To overcome the limitations with respect to the modelling of nucleation mode particles in the early phase of plume dispersion, we present a new framework for the simulation of the particle size distributions on the scales between the exit of the ship stack and the immediate surroundings of ships in the vicinity of harbors. The novel aspects of our model framework include: (1) The use of real world data of ship positions, ship emissions partially based on measured data, measured meteorological data, and measured air pollutant concentrations; (2) allowing for the plume rise of the ship emissions; (3) coupling of gas-phase chemistry with aerosol dynamics; and (4) simultaneous modelling of particle number distributions and their chemical compositions.

The present study is a part of the SNOOP (Shipping-Induced NO_X_ and SO_X_ Emissions-Operational Monitoring Network) project, which aims to find out how ship exhaust emissions affect the marine environment and human health in harbor areas. Measurement campaigns have previously been carried out within the harbor area in the city center of Helsinki (Finland) and along the shipping route in the city of Turku (Finland) in 2010–2011, during winter and summer conditions [11]. The specific objectives of this work are: (i) To trace the evolution of the particle size distribution in the exhaust plumes of passenger ships between the point of release (exit of the ship stack) and the urban area, and (ii) to estimate the exposure to ultrafine particles attributed to shipping in such regions of the harbor cities that are most affected by the ship plumes.

## 2. Materials and Methods

**Figure 1:**
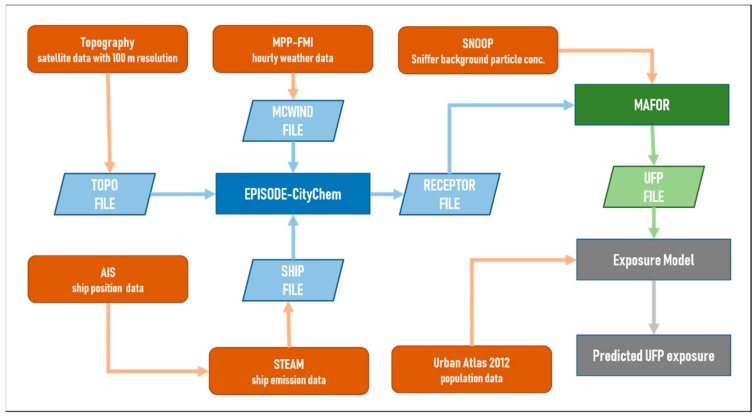
Outline of the sequential processing chain developed in this study. The brown and blue colors refer to the various input datasets and their initial processing, and the atmospheric dispersion and transformation modeling, respectively. The modeling of the aerosol processes and the exposure modeling together with the final product are presented with green and grey colors.

A sequential processing chain was developed in this study consisting of the 3-D chemistry transport model EPISODE-CityChem [33], the multicomponent aerosol dynamic model MAFOR [34,35], and a time–microenvironment–activity model, to assess the short-term population exposure to ultrafine particles originated from ship exhausts. The dilution of aerosol particles along the ship plume centerline was calculated using the output of the EPISODE-CityChem model in a circular grid of modeled receptor concentrations. The aerosol dynamics model uses the obtained dilution parameters to compute the particle number and mass distributions as a function of the distance from the ship stack along the centerline of the ship plume, which corresponds to the time-averaged wind direction of a Gaussian plume. The conceptual outline of the applied methodology is presented in Figure 1.

A drawback of the used approach is that the spatial and temporal heterogeneity of particle concentrations within the ship exhaust plume cannot be represented, because the sub-grid Gaussian dispersion models embedded in the chemistry transport model do not resolve turbulence-induced variations in the concentration fields and have limitations in resolving fine-scale flow structures. Due to the non-linear relationship of particle transformation rates and concentrations, this could cause the aerosol model to either over- or underpredict the rate of aerosol processes in the unstable boundary layer. Nevertheless, the overall model chain allows for a treatment of the aerosol processes in the plume that is consistent with the dispersion of the ship plume exhaust. A clear advantage of the adopted approach is that the uncertainty of the entire model chain is largely minimized through the parameterization with real world observations.

The simulation time of the full model chain for one ship event was approximately 1 h. Simulations were performed on a Linux workstation; Dell Precision T3610 (Intel^®^ Xeon (R) CPU E5-2637 v3 at 3.50 GHz with 64 GB of RAM) (Dell Inc., Round Rock, TX, USA). The computational times were 26 min for EPISODE-CityChem (1-h simulation), 3.2 min for MAFOR (1-h simulation), and 27 min for the exposure model (1-day simulation).

### 2.1. Measurements in the Ship Plume

The measurements of ship emissions were performed in two different harbor environments in Finland: At the harbors in the Helsinki downtown area, and along the shipping channel in the city of Turku (Figure S1, Supplementary Materials). The Viking Line ships operate daily between Helsinki and Stockholm, and Helsinki and Tallinn from the Katajanokka terminal (sites H1 and H2 in Figure S1). The Tallink Silja Line ships operate daily from the Olympic terminal (site H4) between Helsinki and Stockholm. Furthermore, ships of Tallink Silja Line and Viking Line operate daily between Turku and Stockholm departing from Turku Harbor. All of these ships are passenger ships but they also provide roll-on roll-off service (ROPAX ships) and many cars and trucks travel on them. Technical details of the engines, after-treatment systems, etc. are reported in Pirjola et al. [12]. The wintertime campaigns were conducted in Helsinki on 18–29 January 2010 and 7 January–25 February 2011, and in Turku on 2–28 February 2010 and 7–17 February 2011. The summertime campaigns were performed on 26 July–6 August 2010 and 25 July–15 September 2011 in Helsinki, and on 9–19 August 2010 and 1–10 August 2011 in Turku.

Monitoring of the ship plumes was conducted with a mobile laboratory van, “Sniffer” [12]. Sniffer was parked at the measurement sites (H1, H2, and H4 in Helsinki, and T2, T4, T6, T7, and T8 in Turku), facing the ship plume. The sampling occurred at a 2.4-m height from the ground level, above the van’s wind shield. The particle number concentration and size distribution of 7 nm–10 mm particles were measured with an ELPI (Electrical Low Pressure Impactor, Dekati Ltd., Kangasala, Finland) equipped with a filter stage and an additional stage designed to enhance the particle size resolution for UFP [36,37,38]. Gaseous concentrations, such as carbon dioxide (CO_2_), carbon monoxide (CO), nitrogen oxide (NO_X_), and SO_2_, were also measured; however, in this study, we used only the NO_X_ data monitored by an APNA 360CE analyzer (Horiba) to evaluate the modeled dilution curves. A weather station on the roof of the van at a height of 2.9 m above ground level provided measurements of the temperature and relative humidity as well as wind speed and direction, and a global positioning system archived the van’s location. All data were monitored at a 1-s time resolution. All instruments were zero-checked and synchronized before the start of the measurements. The NO_X_ analyzer was calibrated in the NO measurement range 0−2000 ppb, and the used span gases were 0, 200, and 805 ppb of NO. More details can be found in Pirjola et al. [12]. The uncertainty of the measurement results by the ELPI has been estimated to be some tens of percent [39], and the accuracy of the ELPI roughly 25% [40,41]. The uncertainty of the measurement results by the NO_X_ analyzer is approximately 10%.

Ship events in this study were defined as the time periods of ship arrival or ship departure in the harbor when the ship plume could be detected by the instruments of Sniffer. The time periods and the average meteorological conditions for the events in Helsinki and Turku are listed in Table 1. In this study, the Viking Line ships operating in Helsinki are called A and D, and those operating in Turku I and J as in Pirjola et al. [12]. Consistently, the Tallink Silja Line ships operating in Turku are called H, and Tallink ships B and K in Helsinki and Turku, respectively.

### 2.2. Ship Emissions

The Ship Traffic Emission Assessment Model (STEAM) [42,43,44] was used to obtain ship emission data during the ship events in the harbor area. The movement of ships during arrival or departure was tracked by the Automatic Identification System (AIS) data. Ship engine data together with load-dependent emission factors and fuel consumption was used to calculate ship emission data approximately every 10 s during passage of the ship from/to the harbor (within a distance of ca. 4 km).

Major challenges for port emission inventories are the uncertainties of the emissions from the auxiliary engines (AEs) and the boiler fuel consumption. STEAM determines the propulsion engine power as a function of speed and makes certain assumptions about the power profiles of maneuvering ships and ships at berth. The electricity need of each vessel was based on the operation mode, vessel type, and its cargo space description. For vessels carrying passengers, AE power need was linked to passenger capacity as described in our earlier work [43,44]. Regardless, significant uncertainty may be involved in the evaluation of AE and boiler usage because these are usually linked to vessel size without considering primary consumers of electricity onboard.

Data on the ship exhaust characteristics were based on measurements on-board a large ROPAX vessel equipped with a Selective Catalytic Reduction (SCR) system, carried out by the Kymenlaakso University of Applied Sciences during the 2010 campaign. Identical respective values for the exhaust gas temperature and exit velocity were applied to all ships in the dispersion modeling. Table S1, Supplementary Materials, lists the relevant stack parameters, exhaust parameters, and ship geometry.

We applied the emission factors for particle numbers measured by Moldanová et al. [27] for a main engine (ME) burning HFO with FSC = 1 wt % at a 30% engine load (S1_HFO1%_ME-low in [27]). We further assumed that NO_X_ is emitted as 90% NO and 10% NO_2_, based on the recommendation of CIMAC [45]. Furthermore, we assumed that almost all SO_X_ from ships were emitted as SO_2_, i.e., sulfur in the fuel was oxidized to SO_2_, and only 1% was directly released in the form of SO_3_, corresponding to the lower limit of the reported conversion rates (1%−3%) for low sulfur fuels [46]. In addition, we did a sensitivity test using a higher SO_X_-to-SO_3_ conversion rate. The average ship emission of NO_X_, SO_X_, and total particle number (PN) for ships’ events in Helsinki is given in Table S2, Supplementary Materials.

### 2.3. Setup of a 3-D Chemistry Transport Model

The urban-scale chemistry-transport model EPISODE-CityChem [33] was applied to determine the dispersion of the ship plume during the events. In order to resolve pollutant dispersion in the proximity of point sources and lines sources, EPISODE-CityChem combines a 3-D Eulerian grid model with sub-grid Gaussian dispersion models, allowing computation of pollutant concentrations near pollution sources with a high spatial resolution. The advection/-diffusion equation in the Eulerian grid model component for the time-dependent concentration of a chemical species i is given by:

∂c_i_/∂t = −(∂(uc_i_)/∂x + ∂(vc_i_)/∂y + ∂(wc_i_)/∂z) + ∂/∂x(K^(H)^ ∂c_i_/∂x) + ∂/∂y(K^(H)^ ∂c_i_/∂y) + ∂/∂z(K^(z)^ ∂c_i_/∂z) + R_i_(c_1_,…,c_N_) – S_i_(x,y,z,t), (1)

where c_i_ is the mass concentration; u, v, and w are the three wind components of the wind vector; K^(H)^ and K^(z)^ are the horizontal and vertical eddy diffusivities, respectively; R_i_ represents the source terms; and S_i_ represents the sink terms. The terms describing the turbulent diffusion are represented according to mixing length theory (K-theory). The wind velocity field applied in Equation (1) is required to be mass consistent, which is approximated by demanding the wind flow to be divergence free. The mixing length representation of the turbulence terms is valid within this approximation.

The numerical solution employs operator splitting to solve the atmospheric processes on the Eulerian grid: Advection, diffusion, chemical reaction, and deposition. More details on the employed solutions and discretization schemes for the operators are reported by Karl et al. [33] and Hamer et al. [47]. The numerical model time step of the EPISODE-CityChem model is calculated dynamically based on the critical time steps associated with the solution of the horizontal advection, vertical advection, and diffusion processes. The typical model time step in the current model configuration was ~20 s, with horizontal advection in the y-direction being the most critical operator. All operations were performed at an even number of times so that every other operator sequence may be a mirror in the opposite direction of the first sequence to reduce time-splitting errors.

The ship emission data for each 10-s ship position during the ship passage was averaged over 1-min intervals. From the 1-min averages, the ship position at the time when Sniffer recorded the peak concentration of the ship plume was selected as a single point source for the dispersion study. The heat release from ship exhaust stacks represents an additional buoyancy flux that may result in plume rise close to the source. Plume rise of the ship exhaust due to momentum and buoyancy is calculated in the model using the algorithm of Briggs [48,49], which considers different boundary layer (BL) stability conditions characterized by the inverse Monin–Obukhov length (L_O_^−1^). For situations where the exhaust gas temperature exceeds the air temperature, buoyancy is assumed to be dominant. Buoyancy-driven plume rise will affect the final plume height in different ways depending on the BL stability conditions, and therefore the algorithm considers different parameterizations for either unstable and neutral conditions, or stable conditions. The effects of stack downwash and the building-induced disturbances of the flow due to the ship’s building geometry further modify the plume rise. A review of several plume rise studies [50] suggested that plume rise is overestimated by the Briggs equations. In contrast, a comparison by Karl et al. [51] revealed that the implemented scheme gives a lower final plume rise from an elevated points source than two simpler plume rise schemes for all tested atmospheric stability conditions.

The emission height is offset by the final plume rise, giving the effective emission height, H_eff_, where the plume is injected into the corresponding vertical layer of the Eulerian grid. Subsequently, the Gaussian-segmented plume model SEGPLU treats the emission as a temporal sequence of instantaneous releases of a specified pollutant mass. The finite length plume segments are emitted at discrete time intervals, ∆T, given by ∆T = 3600 (s)/2n, where n is an integer value dependent on the meteorological conditions and becomes larger as the wind speed increases. It has been shown that the heat flux at the ship stack has little impact on the in-plume temperature, because it drops quickly to the background air temperature within the first second after release [52]. Therefore, we neglected the impact of the exhaust heat on the plume temperature.

The 3-D simulations of plume dispersion were performed with EPISODE-CityChem on a 15 × 15 km^2^ domain using a 200-m horizontal resolution and 13 vertical layers, where the four lowest layers had a depth of 10 m. For estimating the error of the spatial discretization, a grid resolution test was performed using three levels of grid refinement (cell sizes: 500, 300, 200, and 100 m). The level of grid convergence was evaluated using the grid convergence index (GCI). The GCI provides an estimate of the amount of discretization error in the finest grid solution relative to the converged numerical solution [53], and is defined by:

GCI_21_ = F_s_ ∙ (e_21_/(r_21_^p^−1), (2)

where F_s_ is a safety factor (set to 1.25) multiplying the relative error term; e_21_ is the relative error between the two finest grids; p is the order of convergence; and r is the grid refinement ratio. The grid refinement test showed that the converged numerical solution for pollutant concentrations on the Eulerian grid has an error band of GCI_21_ = 6%, with a 95% confidence level. A reduction of the cell size below 300 m does not change the simulation results substantially.

**Figure 2:**
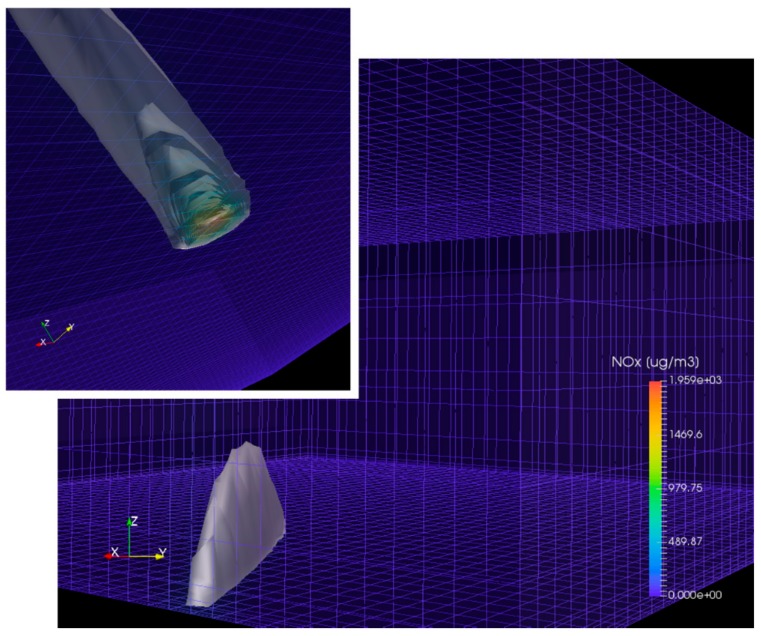
Example numerical results of the simulated ship plume dispersion of ship event J_20100811 using the EPISODE-CityChem model. Larger panel: the shape of the 3-D ship plume within a subset of the model grid. Inserted smaller panel in the upper-left-hand corner: the view of the same ship plume as seen upwards from below the ground surface. Colored contours at the surface indicate the modeled hourly mean concentration of NO_X_ (μg·m^−3^) in the ship plume. Blue lines illustrate the Eulerian 3-D model grid structure.

The model produces output only once every hour; the computed plume dispersion should therefore be considered to be an average steady state. Figure 2 illustrates the shape of the simulated ship plume in the 3-D model for ship event J_20100811.

Meteorological data for Helsinki and Turku was used to parameterize the simulations. Hourly meteorological data for the two domains was estimated using the meteorological preprocessor MPP-FMI [54]. MPP-FMI estimates for the Helsinki domain are based on meteorological measurements at Vantaa airport (60.3267N; 24.95675E) and the weather mast Harmaja (60.10512N; 24.97539E). MPP-FMI estimates for the Turku domain were derived from measurements at met stations Artukainen (60.45439N; 22.1787E) and Rajakari (60.37788N; 22.0964E). The diagnostic model MCWIND [47] was used to calculate divergence-free hourly 3-D wind flow fields based on wind data from two sites. The influence of the moving ship (shear-driven mixing) on the ground wind-flow field was neglected.

Background air concentrations of ozone (O_3_), NO, NO_2_, and PN at the domain borders were based on Sniffer measurements before the respective ship event. The photostationary approximation (PSSA) of the NO_2_-NO-O_3_ reaction cycle was activated. A circular receptor grid (divided into 15° sectors) with Sniffer in the center was used to sample NO_X_ during the simulation at locations near the ship passage.

### 2.4. Aerosol Dynamics Model

Since EPISODE-CityChem currently does not consider aerosol dynamics processes, we applied the aerosol dynamics model MAFOR version 1.9 [34,35], which is able to produce the mass and composition size distributions of a multicomponent aerosol along an air parcel trajectory. MAFOR is a sectional aerosol model that includes gas-phase chemistry; formation of thermodynamically stable clusters by different nucleation mechanisms; condensation of H_2_SO_4_, water (H_2_O), and various organic vapors onto particles, considering molecular dimensions; Brownian coagulation; and mixing with background air. The time evolution of the particle number and mass concentration distribution is solved using the fixed sectional method. The fixed sectional method is computationally efficient and advantageous when treating continuous nucleation, which is important for the modelling of new particle formation. Numerical diffusion in the treatment of condensation/evaporation is negligible due to the use of a high number of size sections (i.e., 120).

The analytical predictor of condensation scheme [55] was employed to calculate the mass transfer of gas molecules to particles and a semi-implicit solution was applied to coagulation. The discrete equations describing the change of the particle number concentration with time were solved with forward finite differences. More details on the numerical methods can be found in Karl et al. [34]. The composition of particles in any size bin can change with time due to multicomponent condensation and/or due to coagulation of particles. MAFOR uses a time step of 0.1 s for the calculation of aerosol dynamics, which is sufficiently small, when compared to the typical time scales in the range 0.5−4 s for dilution in exhaust plumes [56].

In this study, the numerical simulations included two low-volatility organic vapors represented by the n-alkanes C_22_H_46_ and C_28_H_58_ and one extremely low-volatility vapor represented by C_34_H_70_ (a constituent of the lubrication oil). The vapor pressure of n-alkanes as a function of the temperature was adopted from [57]. In addition, the vapor pressure of the n-alkanes is modified by their molar fraction in the particle phase, according to Raoult’s law, and by their molar volume and surface tension according to the Kelvin effect. We chose classical binary homogeneous H_2_SO_4_-H_2_O nucleation (BHN) as the nucleation mechanism in accordance with earlier studies in urban pollution plumes [58]. The BHN parameterization [59,60] used in this study is suitable for predicting the particle formation rate at high temperatures in exhaust conditions by extending the applicable temperature range to 400 K. The kinetic pre-processor KPP v2.2.3 (Virginia Polytechnic Institute and State University, Blacksburg, VA, USA) [61] is used to transform the chemical equations into program code for the gas-phase chemistry solver. The differential equation system of gas-phase reactions is solved with Rosenbrock 3 using automatic time step control. Simulations with MAFOR were initialized with the particle size distributions and mass fractions reported for fresh exhaust at a low engine load (see Section 2.2; [27]).

We selected ship event A_20110912 as a reference to normalize the initial particle size distribution, because A_20110912 is closest to the ship exhaust measurements by [27] in terms of engine type, power, and engine load. Ship plume simulations with MAFOR started one second after release from the ship stack’s exit. In total, we simulated 900 s (15 min) to follow the evolution of the aerosol until the particle concentrations nearly reached the background levels.

The chemical composition of the size-fractionated particles in background air was taken from the LIPIKA study in Helsinki [62]. The chemical composition of ship exhaust particles (estimated based on [27]) and of the background particles is presented in Tables S3 and S4 (Supplementary Materials), respectively. The nucleation mode of the exhaust size distribution (Nuc_2_) was composed of liquid H_2_SO_4_ and primary organic matter with a mass ratio of 20:80. Sensitivity tests were performed regarding the participation of organic substances in the nucleation process as well as regarding the mass composition of the exhaust nucleation mode.

### 2.5. Population Exposure

We estimated the short-term population exposure to ultrafine particles in ship plume peaks in Helsinki and Turku. Population exposure estimates can be used in epidemiological studies to evaluate the health risks associated with outdoor air pollution. Population exposures are generally defined as the pollutant concentration values in environments where people spend their time, taking into account the amount of time they spend within such environments. In dynamic approaches to exposure estimations, the population activity data is used to account for the diurnal variation in population numbers in different locations of the urban area. Therefore, we used a time–microenvironment–activity model [63] to assess the time-variant exposure of the population in a microenvironment (ME). A microenvironment is defined as the location or area with a relatively uniform pollutant concentration, such as the home or the workplace.

In the present study, the focus is on the indoor environments, because we are interested in the exposure in residential areas close to the harbor (ME_home_). Thus, to account for the infiltration of outdoor air pollution into the indoor environments, we applied infiltration factors [63,64]. Infiltration factors (F_inf_) for different pollutant species and different indoor environments are mostly derived from measurements and defined as:

F_inf_ = C_ai_/C_a,_ (3)

where C_ai_ is the indoor air concentration of species a originating from ambient air, and C_a_ is the outdoor air concentration of species a. The infiltration factor, F_inf_, can then be considered specifically for each microenvironment in the calculation of population exposure. Population exposure can also be interpreted as a weighted sum of concentrations, in which the weights are equal to the time spent in each microenvironment, and therefore be written as:

E_i_ = ∑^j^ F_inf,j_ ∙ ∑^t^ C_i,t_ × P_i,j,t_, (4)

where E_i_ is the total exposure in all microenvironments of area i, F_inf,j_ is the infiltration factor for a microenvironment j, C_i,t_ is the pollutant concentration in area i at time t, and P_i,j,t_ is the number of people in the area attributed to the microenvironment at a given time. The infiltration factor, F_inf_, for UFP in home environments in Helsinki and Turku was set to a value of 0.56, which is a mean of the infiltration factors for UFPs from different experimental studies in the urban residential indoor environments of cities in Northern Europe as compiled in a review by Chen and Zhao [65].

Besides the applied microenvironment-based approach, more sophisticated methods for the simulation of building infiltration based on computational fluid dynamics (CFD) models have been published recently [66,67,68], surpassing the need to assume well-mixed pollutant concentrations within the microenvironments. CFD modeling can take into account complex building structures, ventilation parameters, wind pressures acting on the exterior of the building, and buoyancy effects induced by the different indoor and outdoor air temperatures. Nevertheless, we decided to apply a microenvironment-based approach to model indoor exposure due to the high computational costs associated with CFD simulations of a large computational domain, and the lack of specific data in terms of building structure and ventilation parameters.

In this study, the time activity data for the Helsinki metropolitan area in four microenvironments (home, workplace, traffic, and other activities) were adopted from a previous exposure model study [64]. The static population data in Helsinki and Turku was set up as a grid of inhabitants per 100 × 100 m^2^, based on population density data from population statistics in the Urban Atlas 2012 dataset [69].

## 3. Results and Discussion

### 3.1. Dilution Curves between Ship Stack and Ambient

Modeling studies of ship exhaust effects have to deal with the emission process, from the ship funnel to background air via in-plume chemistry and aerosol dynamics and background/plume mixing processes. Those entrainment processes impact in-plume transformation beyond the simple exchange/dilution effects due to the highly nonlinear reaction rates of gases and coagulation rates of particles. Previous studies on the dispersion and transformation of aerosol particles in ship exhaust plumes are scarce [32]. Stuart et al. [70] explored the plume dispersion of ships specially designed for the emission of sea-salt particles employed in marine cloud brightening geoengineering. They used a multi-shelled Gaussian plume model including size-resolved coagulation and found that both plume expansion and coagulation affect the number concentrations of sea-salt particles that were emitted as a single lognormal mode.

We assumed a two-stage dilution process for the dispersion of the ship plume. In the initial high dispersion regime, the freshly released exhaust undergoes fast cooling and the initial plume volume, determined by the stack diameter, expands quickly. This initial expansion ends at the time of 1 s after plume release when the plume cross-section has typically reached a horizontal and vertical extent of 10 and 5.5 m, respectively [71]. The initial stage is followed by a steady-state dilution regime that occurs at a characteristic dilution time scale mainly controlled by the atmospheric turbulence.

#### 3.1.1. First Stage Dilution

We describe the first stage dilution by the jet plume model developed by Vignati et al. [72] for vehicle exhaust plumes, which takes into account both atmospheric turbulence and the entrainment of fresh air due to the jet effect of the exhaust gas. The cross-sectional area of the plume is [72]:

S(t) = ((S_0_)^½^ + t∙σ_w_(0))^2^−(t∙αV_S_)^2^, (5)

where S_0_ is the cross-sectional areas of the ship funnel and V_S_ is the exhaust gas velocity. The entrainment velocity, σ_w_, is given by:

Σ_w_^2^(t) = (αU)^2^ + σ_wt_^2^ + (αu_jet_(t))^2^, (6)

where U is the mean wind speed during the event, σ_wt_ is the traffic-generated turbulence, u_jet_ is the plume jet velocity, which we set equal to V_S_, and α is a proportionality constant that relates wind speed to mechanically created turbulence, here set to 0.1. The traffic-generated turbulence can be ignored for the ship events. Following [71], we assumed that the first dilution stage ends after 1 s. The dilution ratio, DR, between stack emission and the point where atmospheric dilution starts to dominate, is then:

DR = S(1s)/S_0_. (7)

Using Equations (1) and (2), we find DR to be ~8 for all ship events. The temperature in the initial plume, T_P_, changes approximately as [73]:

T_P_−T_A_ = 1/DR∙(T_E_−T_A_), (8)

where T_E_ and T_A_ are the exhaust and ambient air temperatures, respectively. For an ambient temperature of 280 K and T_E_ = 580 K, the plume temperature is ca. 318 K after the end of the initial dilution stage.

The coagulation of particles will compete with the dispersion in the expanding plume. We used the parameterization presented in [70] to calculate the fraction of particles remaining after the initial dilution stage due to coagulation in the super-linearly expanding plume. For details, see Appendix A. The fraction remaining was in the range 0.77−0.93 for most ship events. It was close to 0.5 for ship events with weak wind. Compared to dilution, which results in a fraction remaining of 0.125, coagulation is considered a minor competing process. Neglecting coagulation during the initial dilution stage causes an error of 10% to 15% in the current approach.

#### 3.1.2. Second-Stage Dilution

The second-stage dilution is mainly dependent on atmospheric turbulence [73]. We used a simple horizontal dilution parameterization, following a single-term power series, y(x) = a x^−b^ [10,30], to fit the modeled concentrations in the ship plume, where x (in m) is the downwind distance from the ship stack. In this simple dilution model, parameter a represents the initial concentration at the stack and parameter b describes how rapidly the concentration in the plume declines. The model data on NO_X_ concentrations at the time of the ship event was extracted from the output of the circular receptor grid (embedded in the simulation with EPISODE-CityChem) along the visually determined plume centerline. For the dilution curves, concentration data was ordered by increasing distance from the point of maximum concentration near the ground level, C_max_.

**Figure 3:**
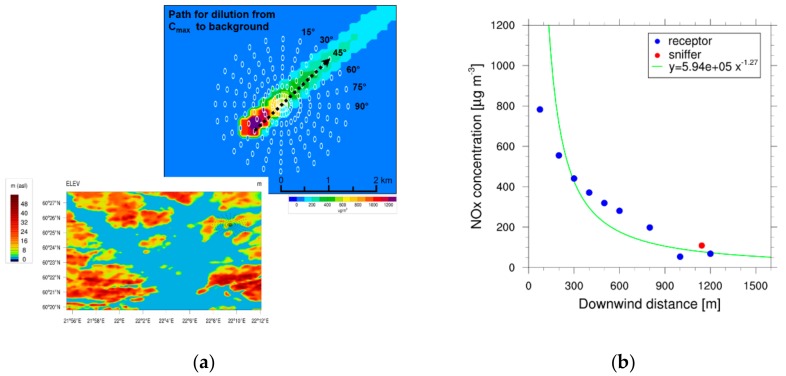
Dilution of the ground concentrations of NO_X_ near the ground level in the ship plume for ship event J_20100811. (**a**) In the upper panel, the circular receptor grid (shown as white circles filled with the color corresponding to the range of concentrations) was used to extract concentration data for the modeled dilution curve. The concentrations were evaluated along a visually determined plume centerline, with increasing distance from the maximum concentration at the ground level to the urban background concentration (shown by the black arrow). The lower panel shows the elevation map of the Turku domain. (**b**) Power-law fit (green line) to the modeled concentrations at receptors (blue dots) in the plume, together with the measured peak 1-min averaged concentration at Sniffer (red dot). Background concentrations were subtracted from the NO_X_ concentration data.

The procedure is illustrated in Figure 3a. Fits to the decaying NO_X_ concentrations, evaluated using the EPISODE-CityChem model, were used for determining the dilution parameters since NO_X_ can be regarded as an inert tracer for which dilution is the only relevant process in the atmosphere. The power-law fit for the example of J_20100811 is shown in Figure 3b while dilution curves for the other events are given in Figure S2 in the Supplementary Materials. Relevant parameters for the ship plume dispersion and results for the empirical dilution curves (a,b) as determined for Helsinki and Turku are listed in Table S5 in the Supplementary Materials. For NO_X_ dilution, parameter b was in the range of 0.99 to 1.47, with an average of 1.26. There was no clear relation between parameter b and the prevailing stability conditions; however, values were higher than the average in unstable conditions and lower than the average during very stable conditions.

### 3.2. Aerosol Dynamics Modeling

MAFOR was applied as a one-dimensional model with downwind distance from the ship stack as the spatial coordinate. The dispersion of particles in the ship plumes was approximated with the simplified treatment of the dilution of particles detailed in Section 3.1.2. The model follows an air parcel in time along the centerline of the ship plume, which corresponds to the time-averaged wind direction of a Gaussian plume. An actual plume may have higher and lower concentration regions, and because of the quadratic relationship of the coagulation rate with particle concentrations this could cause the aerosol model to under predict coagulation in unstable conditions. The particle evolution beginning at time t_0_, i.e., after the end of the first-stage dilution (i.e., at the time of 1 s), is simulated with MAFOR. In addition to nucleation, condensation, and coagulation of particles, dry deposition of particles, and gas-phase chemistry within the plume, the model treats the mixing of the air parcel with gases (NO, NO_2_, SO_2_, and O_3_) and particles from the background air. During the first dilution stage, the in-plume temperature is expected to drop to a value close to the ambient air temperature. The initial temperature of the simulations was thus set to the average air temperature of the event. The equations that govern the time evolution of the particle size distribution in terms of the numbers and mass (of the respective aerosol components) are given in [33] (Equations (16)−(20) therein). In this study, the time evolution was modified by a term that considers the change of the particle number concentration, N_i_, in each size section i by dilution with background particles.

#### 3.2.1. Model Treatment of Dilution

To model the dilution process, we assumed a circular cross-section of the plume. The time-dependent plume height, H_P_, was calculated as:

H_P_(t) = (H_P,0_^2^ + (a’∙(10^−3^∙Ut)^b^)^2^)^½^, (9)

where H_P,0_ is the initial plume height, corresponding to the height of the plume after the first dilution stage, here set to a value of 5.5 m [71]. The dilution parameter, b, is derived from the power-law fit to the empirical dilution curves. Parameter a’ relates to the vertical dispersion parameter, σ_z_, of the Gaussian plume as a function of x (which is represented here in the form of the empirically determined power-law expression, σ_z_ = a’∙x^b^) and depends on the atmospheric stability and the averaging time. The values of a’ are 110.62, 86.49, and 61.14 for unstable, neutral, and stable conditions, respectively [74]. The change of N_i_ (cm^−3^) due to dilution with background air is then:

dN_i_/dt|_dilution_ = −(b/t)∙(N_i_−N_BG,i_), (10)

where N_BG,i_ is the number concentration of background particles in the same size section. It was further assumed that the air parcel is not further influenced by emissions during its travel.

#### 3.2.2. Evolution of the Particle Size Distribution

The evolution of the exhaust particle size distribution as a function of time was computed with the aerosol model, following the air parcel with increasing distance downwind of the ship stack. The initial concentrations of gases in the plume were obtained by dividing the 1-min averaged emission rate (given in Table S3, Supplementary Materials) with the volume flow and multiplying the calculated stack concentration by the dilution ratio. PN concentrations in the diluted exhaust, at the beginning of the simulation, were in the range 10^6^–10^7^ cm^−3^. High numbers of nucleation mode (Nuc_2_) particles (peaking at 5−6 nm) were present in the diluted exhaust, with a mean concentration of 3.2 ± 1.0 × 10^6^ cm^−3^ (±1 SD), making up a fraction of 29% of the total particles.

**Figure 4:**
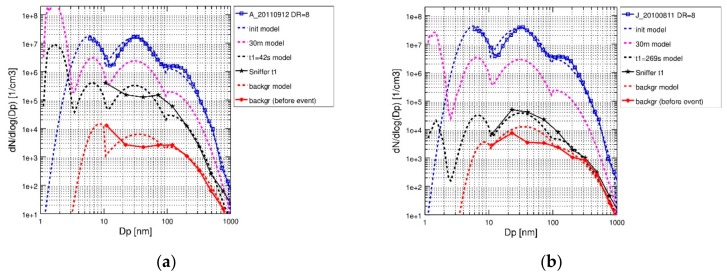
Comparison of the modeled particle size distributions at peak time t_1_ with the particle size distributions recorded at Sniffer; (**a**) Helsinki ship event A_20110912; (**b**) Turku ship event J_20100811. The solid and dashed blue and black lines show the particle size distributions after the initial dilution and at the time t1, respectively. Both events are from the summertime field campaigns. The individual measurements recorded at Sniffer are shown as black stars. Modeled size distribution after a distance of 30 m downwind is indicated as a dashed magenta line. The urban background concentrations are shown as solid and dashed red lines.

Initial concentrations of condensable vapors were estimated, based on the emission rate of SO_X_, to be in the range 0.1−0.5 × 10^11^ cm^−3^, 0.7−3.2 × 10^11^ cm^−3^, 0.7−3.2 × 10^11^ cm^−3^, and 1.2−5.4 × 10^11^ cm^−3^ for H_2_SO_4_, C_22_H_46_, C_28_H_58_, and C_34_H_70_, respectively. Background concentrations of gases and particles were based on 1-min averaged measurements at Sniffer, immediately before the ship passage. Modeled size distributions were compared against the measured size distribution at Sniffer for the time when a peak in the signal of the ELPI instrument was detected that related to the ship passage (measured peak concentration). The time of the peak relative to t_0_ (start time of the simulation) is referred to as t_1_ in the following. Typically, the peak concentrations of PN varied in the range 10^4^–10^6^ cm^−3^, when the ships passed Sniffer at the 200–800 m distance [12]. The initial modeled size distribution was obtained from a fit to the diluted exhaust size distribution (Figure 4, blue squares and line) and the modeled background size distribution from a fit to the measured background size distribution (Figure 4, red diamonds and line).

The temporal evolution of the gas-phase concentrations in the first 15 min of the ship plume is shown in Figure S3, Supplementary Materials. While primary emitted gases, NO, NO_2_, and SO_2_, are diluted to levels in the background air within a few minutes after plume release, background O_3_ quickly entrains the plume, leading to an increase of the concentration of O_3_ in the plume, initially from 10 μg m^−3^ to close to 60 μg m^−3^. In daytime ship events, hydroxyl (OH) radical concentrations forming via photolysis of O_3_ reach levels in the range 1−5 × 10^6^ cm^−3^. The addition of water molecules to the co-emitted SO_3_ causes fast formation of H_2_SO_4_ in the first seconds of the simulation. However, the initial peak of H_2_SO_4_ quickly drops to ambient levels; it is followed by in-plume photochemical formation of H_2_SO_4_ through oxidation of SO_2_ by OH radicals. Maximum H_2_SO_4_ concentration after the first minute ranges between 1−2 × 10^8^ cm^−3^ in the ship plume.

Fresh diesel exhaust particle number size distributions often exhibit a distinct bimodal character, with the corresponding particle types referred to as the nucleation mode and accumulation mode [26,75,76]. The nucleation mode may form in the cooling dilution by homogeneous and heterogeneous nucleation or consist of nonvolatile core particles that formed already in the engine cylinders [75,77,78,79]. The particle number size distribution of the ship exhaust (blue squares in Figure 3) has an additional peak (with a mean diameter at ~40 nm) between the nucleation mode (Nuc_2_, peaking at 5−6 nm) and the accumulation mode. In the simulation of the exhaust, a new nucleation mode (Nuc_1_; peaking at 1.5 nm) consisting of freshly nucleated particles forms rapidly in the size range <3 nm diameter (Figure 4). Nucleation via BHN starts immediately due to the high SO_3_ and H_2_SO_4_ concentrations present in the cooled exhaust. Initial fast nucleation also happens in nighttime events, because no oxidation step is required for conversion of SO_3_ to H_2_SO_4_. Within the first 10 s, at a distance corresponding to 30 m downwind, dilution reduces the particle concentrations by about one order of magnitude. The agreement of the modeled and measured particle size distribution at peak time t_1_ was satisfactory for most ship events, with a tendency to underestimate the measured particle numbers in the size range 70−120 nm in diameter. The comparison between the modeled and measured PN concentrations at peak time t_1_ for all ship events showed that MAFOR was able to predict the number concentration of particles with Dp > 10 nm within ±50% in most ship events (Table S6, Supplementary Materials). Deviations between the modeled and measured PN concentrations are mainly due to the inaccuracies of the derived dilution rates.

#### 3.2.3. Summer–Winter Differences

**Figure 5:**
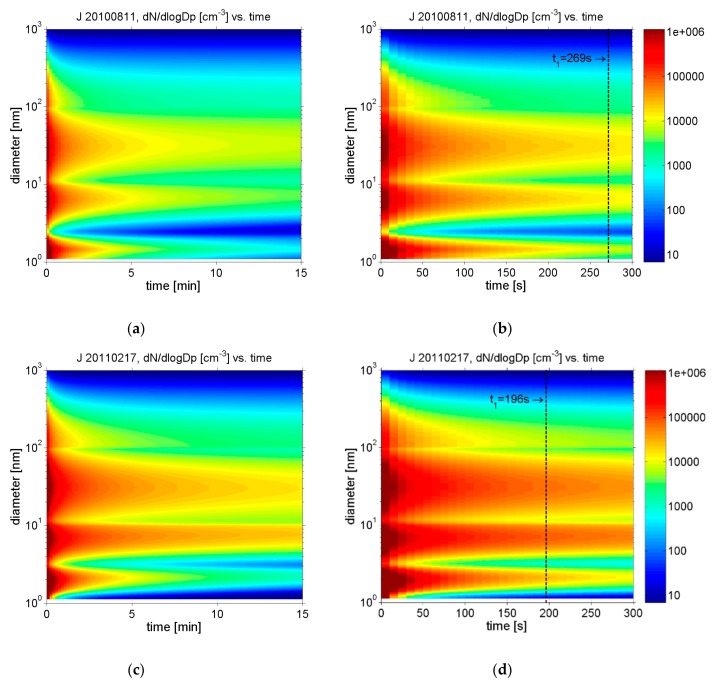
Modelled sequential particle size distributions in the ship plume from 1 s after release from the ship stack up to 15 min downwind (left-hand side panels) and up to 5 min downwind (right-hand side panels). Upper panels: (**a**) J_20100811 in summer, up to 15 min downwind; (**b**) J_20100811 in summer, up to 5 min downwind; lower panels: (**c**) J_20110217 in winter, up to 15 min downwind; (**d**) J_20110217 in winter, up to 5 min downwind. Vertical dashed line indicates the time at which the highest measured concentrations were recorded at Sniffer (t_1_).

Important summer–winter differences are evident in the evolution of the particle size distributions as a function of time, shown as sequential size distribution plots in Figure 5. Stagnant atmospheric conditions in winter ship events (here: J_20110217) led to much slower dilution of the exhaust particles than in the summer ship events (here: J_20100811), with prevailing unstable conditions. Lower temperatures in the winter events favor the condensation of H_2_SO_4_ to particles. Despite the absence of photochemical formation of sulfuric acid in the dark winter, the growth of fresh Nuc_1_ mode particles is much stronger than during the summer event, which had much higher photochemical activity. The growth rate of nucleated particles in the summer event was only 2.0 nm h^−1^ compared to 4.9 nm h^−1^ in the winter event. The enhanced Nuc_1_ growth in winter is partly due to slower dilution of exhaust particles and partly due to higher condensation rates.

#### 3.2.4. Sensitivity Tests on Nucleation and Chemical Composition

We simulated several sensitivity cases to test the assumptions regarding the composition of the exhaust gases and particles and the applied nucleation mechanism:

1. SO_X_-to-SO_3_ conversion rate of 2% (case “2XSO3”);
2. Start temperature of 310 K in the plume (case “T310K”);
3. Nucleation involving organic vapor (details in Appendix B; case “NUHET”); and
4. Nuc_2_ mode composed of 100% liquid H_2_SO_4_ (case “HSULF”).

The sensitivity results for the number concentrations of nucleation mode particles (NP; Dp < 25 nm) and ultrafine particles with Dp > 25 nm, total PN, Nuc_1_ mode diameter, and chemical composition of nucleation mode particles are shown in Figure S4, Supplementary Materials. Doubling the SO_X_-to-SO_3_ conversion rate (case 2XSO3) increases the NP number concentration by 62% after 30 s of travel time. Case T310K shows a similar increase, explained by the BHN parameterization being effective at higher temperatures [60], which gives higher nucleation rates under humid/high sulfuric acid conditions in the temperature range 290−310 K than the low-temperature BHN parameterization [59]. Nucleation between sulfuric acid and organic vapor (case NUHET) gave a slightly higher NP number concentration (by 5%) than nucleation via BHN.

Case HSULF tests the assumption that Nuc_2_ is a volatile nucleation mode that formed in the dilution and cooling of the exhaust. The chemical composition of NP is not sensitive to the assumed composition of Nuc_2_ because the production of extremely low-volatility organic matter (ELV-OM) that occurs in the first second of the simulation overwhelms the contribution of the primary nucleation mode constituents. After 1 s, NP particles are mainly composed of ELV-OM (65% and 67% of the wet particle mass in the base run and HSULF, respectively) and the mass fraction of nonvolatile primary organic matter (NV-POM) that remained is only 6%. Between 1 and 300 s, NP become increasingly hygroscopic due to condensation of H_2_SO_4_, with an increase of attached water mass from 10% up to 21% in the base run (24% in HSULF).

The total dry mass of NP decreases from 33 to 1.1 μg·m^−3^ within 30 s due to dilution and the loss to larger particles by coagulation. Further, following the mass size distribution in the base run with increasing travel time (Figure S5, Supplementary Materials) shows that the primary organic particles of the Nuc_2_ mode disappeared after 120 s. The mass fraction of nonvolatile particles (soot and NV-POM) in UFP in terms of mass remains almost constant at ~50% in the first minutes of the plume travel (Table 2). This is higher than that reported from measurements performed in ship plumes in the port of Gothenburg (24% on average) [80]. We note that the measurement study [80] used unit density to translate from the number to mass, hence assuming a higher density for nonvolatile particles would give a higher nonvolatile fraction. The discrepancy emphasizes the need for size-resolved chemical characterization of UFP in the fresh exhaust from ships.

Differences in the chemical composition of UFPs might be relevant for their associated health impact. Nucleation mode particles will grow increasingly hygroscopic during atmospheric transport. The composition change might be relevant for human health. Water-insoluble particles related to fresh emissions have been associated with high oxidative potential. The oxidative potential is the capacity of particulate matter to carry out or catalyze the formation of reactive oxygen species (ROS) within human lung cells [81,82,83,84]. Studies have linked ROS formed on the surface of soot, such as quinones from oxidation of polycyclic aromatic hydrocarbons by ozone, to increased oxidative potential (e.g., Shiraiwa et al. [85]).

Zhang et al. [86] suggested that exposure to pollutants with high oxidative potential, such as traffic-related pollutants, ultrafine particles, and transition metals, may lead to increased airway oxidative stress and inflammation in elderly adults. Zou et al. [87] observed different cytotoxicity mechanisms of water-soluble and insoluble urban PM_2.5_ at longer exposure times, indicating that solubility should be taken into consideration when evaluating the toxicity of PM_2.5_. McWhinney et al. [88] found that the soot particle surfaces in diesel exhaust contributed the most to the oxidative potential while the contribution of the water-soluble fraction was small. However, it remains to be determined whether the water-soluble and insoluble oxidative potential in the ultrafine particulate matter are linked to differing health end points [89].

### 3.3. Short-Term Population Exposure to Cruise Ship Arrivals/Departures

**Figure 6:**
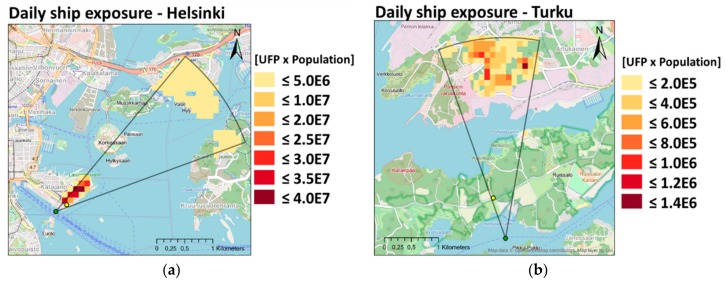
Predicted daily population exposure (cm^−3^ x number of people) in Helsinki and Turku in the microenvironment home during winter: (**a**) Helsinki (based on event A_20110111); (**b**) Turku (based on event J_20110217). Outline indicates the impact area of the ship; green dot marks ship location; yellow dot marks Sniffer location. The results include the estimated exposure from ships, assuming no other emission sources; we also assumed a constant wind direction and a constant urban background UFP concentration.

Short-term population exposure to ship-related UFP in distances up to 3600 m downwind from the ship were calculated based on time-dependent concentrations from MAFOR simulations. To this end, a simple plume representing the ship-impacted area was constructed. Modeled UFP concentrations during a specific ship event in winter (A_20110111 in Helsinki; J_20110217 in Turku) were averaged into 40-m intervals and inserted into circle segments covering a sector of 15° on each side of the plume centerline (concentric circles centered on the ship position at t_1_). Daily mean UFP concentrations were derived based on cruise ship arrivals and departures in Helsinki Katajanokka terminal and in Turku Harbor during the day, assuming the presence of the ship plume during five minutes (based on measured NO_X_ signals at Sniffer) and background concentration during the remaining time. We note that inserting the plume centerline concentration uniformly in the lateral plume cross-section overestimates the concentrations and exposures in the outer part of the plume. Figure 6 shows the daily ship-related exposures in ME_home_ within the ship-impacted area. In Helsinki, daily ship-related exposure is highest in the residential area near the harbor, exceeding the exposure to background UFP by 320%. In Turku, the effect of the ship plume is smaller because densely populated areas are located at a 2.5 km distance from the ship route. Therefore, maximum ship-related exposure exceeds the exposure to background UFP by only 25%. About 650 and 220 persons in Helsinki and Turku, respectively, are living in areas with the highest ship-related population exposures.

The epidemiological evidence is still scarce for the mortality risks of UFP. A multicity study in Central Europe reported positive but not statistically significant associations between prolonged exposures to UFP and respiratory mortality [19]. In contrast to this, a health risk analysis by Breitner et al. [90] conducted in Beijing, China reported a strong association between ultrafine particles and daily cardiovascular mortality with excess risk by 4% per UFP increase of 6250 cm^−3^ in 2-day lagged exposures. Based on the reported strong association in [90], we calculated an excess mortality risk of 15% and 8% for residents that are the most exposed to ship-related UFP in Helsinki and Turku, respectively, which is a factor of 4.5 and 2.2 higher than for residents exposed to typical urban background UFP concentrations in Helsinki (5400 cm^−3^ [91]).

## 4. Conclusions

High concentrations of ultrafine particles have previously been measured in the vicinity of several marine ports in Europe. These concentrations can affect the health of harbor workers and residents living in areas in proximity to the harbors. This paper presented a sequential chain of models, which was used to simulate the temporal evolution of ultrafine particles. We addressed particulate matter originating from the exhaust plumes of ships that arrived or departed from major harbors in Finland in 2010−2011. The modeled dispersion and transformation of particles depends on meteorology, chemical transformation, and aerosol processes. The modeled size distributions in this study were in acceptable agreement with the measured size distributions, at distances of up to several hundred meters from the ship. We draw the following conclusions:

1. We evaluated that the dilution ratio (DR) was approximately eight (confidence interval: 7 to 10) after the initial fast expansion of the exhaust plume. The estimated dilution ratio was smaller than the DR range 10−40, which has been previously reported for ocean-going vessels [52,71] for a plume age of 1 s in the marine boundary layer.
2. The initial cooling and expansion lead to a fully formed nucleation mode (Nuc_2_, peaking at 5−6 nm). The exhaust size distribution [27] that we used as a starting point of the modeling presumably already includes the ceased initial nucleation of the fresh exhaust. On-road measurements showed that the nucleation mode in vehicle exhaust was already present under 0.7 s of residence time in ambient air [92]. The formation of new particles in fresh diesel exhaust by nucleation of H_2_SO_4_ (and organic substances) might even occur during the first milliseconds after release [26].
3. The modeling showed that a partially formed nucleation mode (Nuc_1_; peaking at 1.5 nm) consisting of freshly nucleated sulfate particles and condensed organics was formed within the first few seconds after the initial stage, irrespective of the applied nucleation mechanism (BHN or HET).
4. The Baltic Sea has been an emission control area for SO_X_ (SECA) since 2006. After January 2015, the ships operating there must use reduced-sulphur fuels with FSC below 0.1 wt %. The introduction of these emission reductions has resulted in a substantial decrease of both the primary particulate matter emissions and secondary formation of PM_2.5_ from shipping [93]. The lower sulfur emissions after introduction of the stricter regulations have probably also changed the role of H_2_SO_4_ in the nucleation of ship exhausts.
5. The increased concentrations of UFP attributed to ship plumes significantly increase the short-term exposures of the population in the vicinity of the harbor areas. Due to the frequently travelling ships, these concentrations can substantially affect the daily mean UFP exposures and increase mortality risks. A previous health study in Helsinki [94] found associations between all particle fractions and cardiorespiratory health among people aged 65 years or older; however, according to that study, the association was stronger for the accumulation mode than for ultrafine particles. This highlights the need for epidemiological studies that take into account the spatial-temporal distributions of various particle size fractions, especially those of ultrafine particles, in the assessment of short-term exposures in different age groups.

## Figures and Tables

**Table 1:** Information on the ship events in Helsinki and Turku. Selected average meteorological conditions (air temperature T_A_, relative humidity RH, wind speed U, wind direction WD) are presented during the ship events. The direction of the ship on the route is indicated by: arr. = arrival; dep. = departure.

Event	Measurement Site and Ship Direction	Start Time	End Time	T_A_ (K)	RH (%)	U (m s^−1^)	WD (°)
A_20110111	H2, dep.	11:25:00	11:55:00	271	90	4.3	289
A_20110912	H1, dep.	11:30:00	11:47:00	290	73	6.3	130
A_20110913	H2, dep.	11:20:00	11:45:00	290	82	4.8	213
B_20100803	H7, dep.	18:30:00	18:40:00	292	80	5.4	145
D_20110111	H2, dep.	17:30:00	17:50:00	274	81	5.1	232
D_20110908	H2, dep.	17:25:00	17:50:00	289	78	2.0	187
H_20100811	T8, dep.	08:40:00	08:50:00	292	71	5.5	249
H_20100817	T2, dep.	08:30:00	08:40:00	289	69	3.5	173
I_20100812	T2, arr.	19:18:00	19:35:00	295	61	3.0	233
I_20110207	T7, arr.	19:20:00	19:30:00	272	94	3.6	250
J_20100811	T7, dep.	09:00:00	09:10:00	292	72	4.6	219
J_20100817	T4, dep.	09:00:00	09:10:00	290	65	5.7	47
J_20110217	T2, arr.	07:00:00	07:25:00	258	79	2.4	175
K_20110802	T6, arr.	20:00:00	20:30:00	293	49	0.8	34

**Table 2:** Simulated chemical composition of ultrafine particles (dry weight) in the plume after different travel times. Mass concentration (μg·m^−3^) in top part and mass fraction in bottom part. Components: Soot (corresponding to elemental carbon), ELV-OM—extreme low-volatility organic matter, SV-OM—semivolatile organic matter, NV-POM—nonvolatile primary organic matter.

**Travel Time (s)**	**Soot**	**ELV-OM**	**Sulfate**	**SV-OM**	**NV-POM**
1	41.6	55.6	19.2	8.30	37.8
30	2.46	2.84	1.15	0.57	2.20
120	0.66	0.70	0.33	0.17	0.57
300	0.29	0.28	0.17	0.09	0.23
**Travel Time (s)**	***Mass Fraction (Dry Weight)***				
1	0.26	0.34	0.12	0.05	0.23
30	0.27	0.31	0.12	0.06	0.24
120	0.27	0.29	0.14	0.07	0.23
300	0.27	0.26	0.16	0.08	0.23

## Supplementary Materials

The following are available online at https://www.mdpi.com/1660-4601/17/3/777/s1, Figure S1: Measurement sites in (a) Helsinki South Harbor (sites H1, H2 and H4) and in West Harbor (site H7), and (b) along the shipping channel to Turku Harbor (sites T2, T4, T6, T7 and T8), Figure S2: Power-law fit to the modeled concentrations at receptors in the ship plume based on dispersion simulations with EPISODE-CityChem, Figure S3: Gas-phase concentrations of relevant tracers and atmospheric oxidants in the ship plume in the first 15 min after release, Figure S4: Results from the sensitivity tests with the aerosol dynamics model, Figure S5: Mass composition distribution in the size range 1−600 nm at different travel times of the ship plume simulated with the MAFOR model, Table S1: Ship stack parameters and ship geometry, Table S2: Ship engine power (ME: main engine, AE: auxiliary engine) and ship emissions of NO_X_, SO_X_ and total particle number (PN) based on STEAM during ship events, Table S3: Mass composition (in %) of the ship exhaust particle emissions, Table S4: Mass composition (in %) of particulate matter in the urban background air, Table S5: Conditions of the ship plume dispersion and parameters used in the single term power series fits to the modeled NO_X_ concentration data for ship events in Helsinki and Turku, Table S6: Comparison of modeled and measured PN concentrations at peak time t_1_, for ship events in Helsinki and Turku. The EPISODE-CityChem model is available at https://doi.org/10.5281/zenodo.3549415. The access to the MAFOR model can be granted after signing a collaborative agreement with M.K. The access to the exposure model is available on request to M.R.

## Appendix A. Fraction of Particles Remaining

The change of particle number due to coagulation in the expanding plume of the initial dilution stage can be solved analytically. The number of particles in the expanding plume, N_P_, that is reached asymptotically with increasing time is [95]:

N_P_ = N_P0_∙N_T_/(N_P0_ + N_T_), (A1)

where N_P0_ is the number of particles directly at the stack and the unique dimensionless number, N_T_, which depends only on the coagulation kernel and the plume volume. In a plume that expands super-linearly with time, N_T_ is defined as:

N_T_ = (2∙V_P0_∙(α’−1))/K_C_∙t_i_, (A2)

where V_P0_ is the initial plume volume, t_i_ is the time of the expansion from the stack to its initial volume (here 1 s), and K_C_ is the effective coagulation coefficient. Parameter α’ depends on the atmospheric stability (α’ > 1)). Based on Equations (A1) and (A2), Stuart et al. [70] derive the fraction of particles remaining (due to coagulation) at the end of the initial plume expansion:

F = N_P_/N_P0_ = 2∙(α’−1)/(K_C_∙t_i_∙N_P0_ + 2∙(α’−1)). (A3)

Based on simulations with a multi-shelled Gaussian plume model, Stuart et al. [70] provide a parameterization, where F depends on five parameters: Wind speed (U), stack radius (R_S_), particle number emission rate (E_PN_), particle mean diameter (Dp), and geometric standard deviation (σ_P_):

F = k/((U/U_0_)^a^ + (R_S_/R_S0_)^b^ + (E_PN_/E_PN0_)^c^ + (σ_P_/σ_P0_)^d^ + (Dp/Dp_0_)^e^ + k), (A4)

where a, b, c, d, e, and k are fitted coefficients for different stability classes (Table 2 in [54]). The parameters with “_0_” subscripts refer to the base conditions in [70] and are: U_0_ = 8 m s^−1^, R_S0_ = 1.2 m, E_PN0_ = 1.1 × 10^17^ s^−1^, σ_P0_ = 1.2, and Dp_0_ = 200 nm.

## Appendix B. Nucleation between Sulfuric Acid and Organic Vapors

Paasonen et al. [96] proposed the occurrence of homogeneous homomolecular nucleation of H_2_SO_4_ along with homogeneous heteromolecular nucleation between H_2_SO_4_ and organic vapor molecules (HET), with the following production rate of nucleated particles:

J_nucl_ = K_1_ [H_2_SO_4_]^2^ + K_2_ [H_2_SO_4_] [COV_SV_], (A5)

where the values of the pre-factors K_1_ and K_2_ for HET nucleation in diesel exhaust are 3.8 × 10^−17^ and 5.6 × 10^−17^ cm^3^ s^−1^, respectively, adopted from a study of a diesel engine equipped with an oxidative after-treatment system [30]. Since n-alkanes do not form hydrogen bonds with sulfuric acid, we introduced a semivolatile organic compound, COV_SV_, with the properties of adipic acid that takes part in the HET nucleation. The COV_S_ concentration was 6 × 10^11^ cm^−3^ in the sensitivity run using HET nucleation.

## References

[1] Smith T.W.P., Jalkanen J.-P., Anderson B.A., Corbett J.J., Faber J., Hanayama S., O’Keeffe E., Parker S., Johansson L., Aldous L. (2015). Third IMO GHG Study 2014.

[2] Sinha P., Hobbs P.V., Yokelson R.J., Christian T.J., Kirchstetter T.W., Bruintjes R. (2003). Emissions of trace gases and particles from two ships in the southern Atlantic Ocean. Atmos. Environ..

[3] Agrawal H., Eden R., Zhang X., Fine P.M., Katzenstein A., Miller J.W., Ospital J., Teffera S., Rd C.D. (2009). Primary particulate matter from ocean-going engines in the Southern California Air Basin. Environ. Sci. Technol..

[4] Hunter K.A., Liss P.S., Surapipith V., Dentener F., Duce R., Kanakidou M., Kubilay N., Mahowald N., Okin G., Sarin M. (2011). Impacts of anthropogenic SO_X_, NO_X_ and NH_3_ on acidification of coastal waters and shipping lanes. Geophys. Res. Lett..

[5] Capaldo K.P., Corbett J.J., Kasibhatla P., Fischbeck P., Pandis S.N. (1999). Effects of ship emissions on sulphur cycling and radiative climate forcing over the ocean. Nature.

[6] WHO (2016). Ambient Air Pollution: A Global Assessment of Exposure and Burden of Disease.

[7] Pope C.A., Burnett R.T., Thun M.J., Calle E.E., Krewski D., Ito K., Thurston G.D. (2002). Lung cancer, cardiopulmonary mortality, and long-term exposure to fine particulate air pollution. JAMA.

[8] Corbett J.J., Winebrake J.J., Green E.H., Kasibhatla P., Eyring V., Lauer A. (2007). Mortality from ship emissions: A global assessment. Environ. Sci. Technol..

[9] Sofiev M., Winebrake J.J., Johansson L., Carr E.W., Prank M., Soares J., Vira J., Kouznetsov R., Jalkanen J.-P., Corbett J.J. (2018). Cleaner fuels for ships provide public health benefits with climate tradeoffs. Nat. Commun..

[10] Healy R.M., O’Connor I.P., Hellebust S., Allanic A., Sodeau J.R., Wenger J.C. (2009). Characterisation of single particles from in-port ship emissions. Atmos. Environ..

[11] Keuken M.P., Henzing J.S., Zandveld P., Van den Elshout S., Karl M. (2012). Dispersion of particle numbers and elemental carbon from road traffic, a harbor and an airstrip in the Netherlands. Atmos. Environ..

[12] Pirjola L., Pajunoja A., Walden J., Jalkanen J.-P., Rönkkö T., Kousa A., Koskentalo T. (2014). Mobile measurements of ship emissions in two harbour areas in Finland. Atmos. Meas. Tech..

[13] Lopes M., Russo A., Gouveia C., Ferreira F. (2019). Monitoring of ultrafine particles in the surrounding urban area of in-land passenger ferries. J. Environ. Prot..

[14] Oberdörster G., Oberdörster E., Oberdörster J. (2005). Nanotoxicology: An emerging discipline evolving from studies of ultrafine particles. Environ. Health Persp..

[15] Rückerl R., Schneider A., Breitner S., Cyrys J., Peters A. (2011). Health effects of particulate air pollution: A review of epidemiological evidence. Inhal. Toxicol..

[16] Kendall M., Holgate S. (2012). Health impact and toxicological effects of nanomaterials in the lung. Respirology.

[17] Miller M.R., Raftis J.B., Langrish J.P., McLean S.G., Samutrtai P., Connell S.P., Wilson S., Vesey A.T., Fokkens P.H.B., Boere A.J.F. (2017). Inhaled nanoparticles accumulate at sites of vascular disease. ACS Nano.

[18] Cassee F.R., Héroux M.-E., Gerlofs-Nijland M.E., Frank J., Kelly F.J. (2013). Particulate matter beyond mass: Recent health evidence on the role of fractions, chemical constituents and sources of emission. Inhal. Toxicol..

[19] Lanzinger S., Schneider A., Breitner S., Stafoggia M., Erzen I., Dostal M., Peters A. (2016). Associations between ultrafine and fine particles and mortality in five central European cities - Results from the UFIREG study. Environ. Int..

[20] Kukkonen J., Karl M., Keuken M.P., Van der Gon H.A.C.D., Denby B.R., Singh V., Douros J., Manders A., Samaras Z., Moussiopoulos N. (2016). Modelling the dispersion of particle numbers in five European cities. Geosci. Model Dev..

[21] Petzold A., Hasselbach J., Lauer P., Baumann R., Franke K., Gurk C., Schlager H., Weingartner E. (2008). Experimental studies on particle emissions from cruising ship, their characteristic properties, transformation and atmospheric lifetime in the marine boundary layer. Atmos. Chem. Phys..

[22] Lieke K.I., Rosenørn T., Pedersen J., Larsson D., Kling J., Fuglsang K., Bilde M. (2013). Micro- and nanostructural characteristics of particles before and after an exhaust gas recirculation system scrubber. Aerosol Sci. Technol..

[23] Lyyränen J., Jokiniemi J., Kauppinen E.I., Joutsensaari J. (1999). Aerosol characterisation in medium-speed diesel engines operating with heavy fuel oils. J. Aerosol Sci..

[24] Murphy S.M., Agrawal H., Sorooshian A., Padró L.T., Gates H., Hersey S., Welch W.A., Jung H., Miller J.W., Cocker D.R. (2009). Comprehensive simultaneous shipboard and airborne characterization of exhaust from a modern container ship at sea. Environ. Sci. Technol..

[25] Lack D.A., Corbett J.J., Onasch T., Lerner B., Massoli P., Quinn P.K., Bates T.S., Covert D.S., Coffman D., Sierau B. (2009). Particulate emissions from commercial shipping: Chemical, physical and optical properties. J. Geophys. Res..

[26] Kittelson D.B. (1998). Engines and nanoparticles: A review. J. Aerosol Sci..

[27] Moldanová J., Fridell E., Winnes H., Holmin-Fridell S., Boman J., Jedynska A., Tishkova V., Demirdjian B., Joulie S., Bladt H. (2013). Physical and chemical characterisation of PM emissions from two ships operating in European Emission Control Areas. Atmos. Meas. Tech..

[28] Colom J.M., Alzueta M.U., Christensen J.M., Glarborg P., Cordtz R., Schramm J. (2016). Importance of vanadium-catalyzed oxidation of SO_2_ to SO_3_ in two-stroke marine diesel engines. Energy Fuels.

[29] Reche C., Viana M., Moreno T., Querol X., Alastuey A., Pey J., Pandolfi M., Prévôt A., Mohr C., Richard A. (2011). Peculiarities in atmospheric particle number and size-resolved speciation in an urban area in the western Mediterranean: Results from the DAURE campaign. Atmos. Environ..

[30] Pirjola L., Karl M., Rönkkö T., Arnold F. (2015). Model studies of volatile diesel exhaust particle formation: Are organic vapours involved in nucleation and growth?. Atmos. Chem. Phys..

[31] Babu S.S., Kompalli S.K., Moorthy K.K. (2016). Aerosol number size distributions over a coastal semi urban location: Seasonal changes and ultrafine particle bursts. Sci. Total Environ..

[32] Tian J., Riemer N., West M., Pfaffenberger L., Schlager H., Petzold A. (2014). Modelling the evolution of aerosol particles in a ship plume using PartMC-MOSAIC. Atmos. Chem. Phys..

[33] Karl M., Walker S.-E., Solberg S., Ramacher M.O.P. (2019). The Eulerian urban dispersion model EPISODE. Part II: Extensions to the source dispersion and photochemistry for EPISODE-CityChem v1.2 and its application to the city of Hamburg. Geosci. Model Dev..

[34] Karl M., Gross A., Pirjola L., Leck C. (2011). A new flexible multicomponent model for the study of aerosol dynamics in the marine boundary layer. Tellus B.

[35] Karl M., Kukkonen J., Keuken M.P., Lützenkirchen S., Pirjola L., Hussein T. (2016). Modeling and measurements of urban aerosol processes on the neighborhood scale in Rotterdam, Oslo and Helsinki. Atmos. Chem. Phys..

[36] Keskinen J., Pietarinen K., Lehtimäki M. (1992). Electrical low pressure impactor. J. Aerosol Sci..

[37] Marjamäki M., Ntziachristos L., Virtanen A., Ristimäki J., Keskinen J., Moisio M., Palonen M., Lappi M. (2002). Electrical Filter Stage for the ELPI.

[38] Yli-Ojanperä J., Kannosto J., Marjamäki M., Keskinen J. (2010). Improving the nanoparticle resolution of the ELPI. Aerosol. Air Qual. Res..

[39] Schlatter J. (2010). EURAMET Project 1027: Comparison of Nanoparticle Number Concentration and Size Distribution.

[40] Giechaskiel B., Maricq M., Ntziachristos L., Dardiotis C., Wang X., Axmann H., Bergmann A., Schindler W. (2014). Review of motor vehicle particulate emissions sampling and measurement: From smoke and filter mass to particle number. J. Aerosol Sci..

[41] Salo L., Mylläri F., Maasikmets M., Niemelä V., Konist A., Vainumäe K., Kupri H.-L., Titova R., Simonen P., Aurela M. (2019). Emission measurements with gravimetric impactors and electrical devices: An aerosol instrument comparison. Aerosol Sci. Technol..

[42] Jalkanen J.-P., Brink A., Kalli J., Pettersson H., Kukkonen J., Stipa T. (2009). A modelling system for the exhaust emissions of marine traffic and its application in the Baltic Sea area. Atmos. Chem. Phys..

[43] Jalkanen J.-P., Johansson L., Kukkonen J., Brink A., Kalli J., Stipa T. (2012). Extension of an assessment model of ship traffic exhaust emissions for particulate matter and carbon monoxide. Atmos. Chem. Phys..

[44] Johansson L., Jalkanen J.-P., Kukkonen J. (2017). Global assessment of shipping emissions in 2015 on a high spatial and temporal resolution. Atmos. Environ..

[45] CIMAC (2008). Guide to Diesel Exhaust Emissions Control of NOX, SOX, Particulates, Smoke and CO_2_-Seagoing Ships and Big Stationary Diesel Power Plants.

[46] Zhou S., Zhou J., Zhu Y. (2019). Chemical composition and size distribution of particulate matters from marine diesel engines with different fuel oils. Fuel.

[47] Hamer P.D., Walker S.-E., Sousa-Santos G., Vogt M., Vo-Thanh D., Lopez-Aparicio S., Ramacher M.O.P., Karl M. (2019). The urban dispersion model EPISODE. Part 1: A Eulerian and sub-grid-scale air quality model and its application in Nordic winter conditions. Geosci. Model Dev. Discuss..

[48] Briggs G.A., Englund H.M., Berry W.T. (1971). Some recent analyses of plume rise observation. Proceedings of the Second International Clean Air Congress.

[49] Briggs G.A. (1974). Diffusion Estimation for Small Emissions.

[50] VDI (1985). Ausbreitung von Luftverunreinigungen in der Atmosphäre.

[51] Karl M., Castell N., Simpson D., Solberg S., Starrfelt J., Svendby T., Walker S.-E., Wright R.F. (2014). Uncertainties in assessing the environmental impact of amine emissions from a CO2 capture plant. Atmos. Chem. Phys..

[52] Chosson F., Paoli R., Cuenot B. (2008). Ship plume dispersion rates in convective boundary layers for chemistry models. Atmos. Chem. Phys..

[53] Roache P.J. (1994). Perspective. A method for uniform reporting of grid refinement studies. J. Fluids Eng..

[54] Karppinen A., Kukkonen J., Elolähde T., Konttinen M., Koskentalo T., Rantakrans E. (2000). A modelling system for predicting urban air pollution: Model description and applications in the Helsinki metropolitan area. Atmos. Environ..

[55] Jacobson M.Z. (1997). Numerical techniques to solve condensational and dissolutional growth equations when growth is coupled to reversible reactions. Aerosol Sci. Technol..

[56] Ketzel M., Berkowicz R. (2004). Modelling the fate of ultrafine particles from exhaust pipe to rural background: An analysis of time scales for dilution, coagulation and deposition. Atmos. Environ..

[57] Lemmon E.W., Goodwin A.R.H. (2000). Critical properties and vapor pressure equation for alkanes C_n_H_2n+2_: Normal alkanes and isomers for n = 4 through n = 9. J. Phys. Chem. Ref. Data.

[58] Kerminen V.-M., Wexler A.S. (1996). The occurrence of sulfuric acid-water nucleation in plumes: Urban environment. Tellus B Chem. Phys. Meteorol..

[59] Vehkamäki H., Kulmala M., Napari I., Lehtinen K.E.J., Timmreck C., Noppel M., Laaksonen A. (2002). An improved parameterization for sulfuric acid-water nucleation rates for tropospheric and stratospheric conditions. J. Geophys. Res..

[60] Vehkamäki H., Kulmala M., Lehtinen K.E.J., Noppel M. (2003). Modelling binary homogeneous nucleation of water-sulfuric acid vapours: Parameterisation for high temperature emissions. Environ. Sci. Technol..

[61] Sandu A., Sander R. (2006). Technical note: Simulating chemical systems in Fortran90 and Matlab with the Kinetic PreProcessor KPP-2.1. Atmos. Chem. Phys..

[62] Pohjola M.A., Pirjola L., Karppinen A., Härkönen J., Korhonen H., Hussein T., Ketzel M., Kukkonen J. (2007). Evaluation and modelling of the size fractionated aerosol particle number concentration measurements nearby a major road in Helsinki–Part I: Modelling results within the LIPIKA project. Atmos. Chem. Phys..

[63] Ramacher M.O.P., Karl M., Bieser J., Jalkanen J.-P., Johansson L. (2019). Urban population exposure to NO_X_ emissions from local shipping in three Baltic Sea harbour cities–a generic approach. Atmos. Chem. Phys..

[64] Soares J., Kousa A., Kukkonen J., Matilainen L., Kangas L., Kauhaniemi M., Riikonen K., Jalkanen J.-P., Rasila T., Hänninen O. (2014). Refinement of a model for evaluating the population exposure in an urban area. Geosci. Model Dev..

[65] Chen C., Zhao B. (2011). Review of relationship between indoor and outdoor particles: I/O ratio, infiltration factor and penetration factor. Atmos. Environ..

[66] Argyropoulos C.D., Ashraf A.M., Markatos N.C., Kakosimos K.E. (2017). Mathematical modelling and computer simulation of toxic gas building infiltration. Proc. Saf. Environ. Prot..

[67] Argyropoulos C.D., Hassan H., Kumar P., Kakosimos K.E. (2020). Measurements and modelling of particulate matter building ingress during a severe dust storm event. Buil. Environ..

[68] Liu X., Peng Z., Liu X., Zhou R. (2020). Dispersion characteristics of hazardous gas and exposure risk assessment in a multiroom building environment. Int. J. Environ. Res. Public Health.

[69] Batista e Silva F., Poelman H. (2016). Mapping Population Density in Functional Urban Areas–A Method to Downscale Population Statistics to Urban Atlas Polygons.

[70] Stuart G.S., Stevens R.G., Partanen A.-I., Jenkins A.K.L., Korhonen H., Forster P.M., Spracklen D.V., Pierce J.R. (2013). Reduced efficacy of marine cloud brightening geoengineering due to in-plume aerosol coagulation: Parameterization and global implications. Atmos. Chem. Phys..

[71] Von Glasow R., Lawrence M.G., Sander R., Crutzen P.J. (2003). Modeling the chemical effects of ship exhaust in the cloud-free marine boundary layer. Atmos. Chem. Phys..

[72] Vignati E., Berkowicz R., Palmgren F., Lyck E., Hummelshoj P. (1999). Transformation of size distributions of emitted particles in streets. Sci. Total Environ..

[73] Zhang K.M., Wexler A.S. (2004). Evolution of particle number distribution near roadways–Part I: Analysis of aerosol dynamics and its implications for engine emission measurement. Atmos. Environ..

[74] Petersen W.B. (1980). User’s Guide for Hiway-2: A Highway Air Pollution Model.

[75] Kittelson D.B., Watts W.F., Johnson J.P. (2006). On-road and laboratory evaluation of combustion aerosols–Part 1: Summary of diesel engine results. Aerosol Sci..

[76] Lähde T., Rönkkö T., Virtanen A., Solla A., Kytö M., Söderström C., Keskinen J. (2010). Dependence between nonvolatile nucleation mode particle and soot number concentrations in an EGR equipped heavy-duty diesel engine exhaust. Environ. Sci. Technol..

[77] Seong H., Choi S., Lee K. (2014). Examination of nanoparticles from gasoline direct-injection (GDI) engines using transmission electron microscopy (TEM). Int. J. Automot. Technol..

[78] Sgro L.A., Sementa P., Vaglieco B.M., Rusciano G., D’Anna A., Minutolo P. (2012). Investigating the origin of nuclei particles in GDI engine exhausts. Combust. Flame.

[79] Lähde T., Virtanen A., Happonen M., Söderström C., Kytö M., Keskinen J. (2014). Heavy-duty, off-road diesel engine low-load particle number emissions and particle control. J. Air Waste Manag. Assoc..

[80] Jonsson A.M., Westerlund J., Hallquist M. (2011). Size-resolved particle emission factors for individual ships. Geophys. Res. Lett..

[81] Kumagai Y., Koide S., Taguchi K., Endo A., Nakai Y., Yoshikawa T., Shimojo N. (2002). Oxidation of proximal protein sulfhydryls by phenanthraquinone, component of diesel exhaust particles. Chem. Res. Toxicol..

[82] Li N., Sioutas C., Cho A., Schmitz D., Misra C., Sempf J., Wang M., Oberley T., Froines J., Nel A. (2003). Ultrafine particulate pollutants induce oxidative stress and mitochondrial damage. Environ. Health Perspect..

[83] Cho A.K., Constantinos S., Miguel A.H., Kumagai Y., Schmitz D.A., Singh M., Eigure-Fernandez A., Froines J.R. (2005). Redox activity of airborne particulate matter at different sites in the Los Angeles Basin. Environ. Res..

[84] Li Q., Wyatt A., Kamens R.M. (2009). Oxidant generation and toxicity enhancement of aged-diesel exhaust. Atmos. Environ..

[85] Shiraiwa M., Sosedova Y., Rouvière A., Yang H., Zhang Y., Abbatt J.P.D. (2011). The role of long-lived reactive oxygen intermediates in the reaction of ozone with aerosol particles. Nature Chem..

[86] Zhang X., Staimer N., Gillen D.L., Tjoa T., Schauer J.J., Shafer M.M., Hasheminassab S., Pakbin P., Vaziri N.D., Sioutas C. (2016). Associations of oxidative stress and inflammatory biomarkers with chemically-characterized air pollutant exposures in an elderly cohort. Environ. Res..

[87] Zou Y., Jin C., Su Y., Li J., Bangshang Z. (2016). Water soluble and insoluble components of urban PM_2.5_ and their cytotoxic effects on epithelial cells (A549) in vitro. Environ. Pollut..

[88] McWhinney R.D., Badali K., Liggio J., Li S.-M., Abbatt J.P.D. (2013). Filterable redox cycling activity: A comparison between diesel exhaust particles and secondary organic aerosol constituents. Environ. Sci. Technol..

[89] Fang T., Zeng L., Gao D., Verma V., Stefaniak A.B., Weber R.J. (2017). Ambient size distributions and lung deposition of aerosol dithiothreitol-measured oxidative potential: Contrast between soluble and insoluble particles. Environ. Sci. Technol..

[90] Breitner S., Liu L., Cyrys J., Brüske I., Franck U., Schlink U., Leitte A.M., Herbarth O., Wiedensohler A., Wehner B. (2011). Sub-micrometer particulate air pollution and cardiovascular mortality in Beijing, China. Sci. Total Environ..

[91] Dos Santos-Juusela V., Petäjä T., Kousa A., Hämeri K. (2013). Spatial-temporal variations of particle number concentrations between a busy street and the urban background. Atmos. Environ..

[92] Rönkkö T., Virtanen A., Kannosto J., Keskinen J., Lappi M., Pirjola L. (2007). Nucleation mode particles with a nonvolatile core in the exhaust of a heavy duty diesel vehicle. Environ. Sci. Technol..

[93] Karl M., Bieser J., Geyer B., Matthias V., Jalkanen J.-P., Johansson L., Fridell E. (2019). Impact of a nitrogen emission control area (NECA) on the future air quality and nitrogen deposition to seawater in the Baltic Sea region. Atmos. Chem. Phys..

[94] Halonen J.I., Lanki T., Yli-Tuomi T., Tiittanen P., Kulmala M., Pekkanen J. (2009). Particulate air pollution and acute cardiorespiratory hospital admissions and mortality among the elderly. Epidemiology.

[95] Turco R.P., Yu F. (1997). Aerosol invariance in expanding coagulating plumes. Geophys. Res. Lett..

[96] Paasonen P., Nieminen T., Asmi E., Manninen H.E., Petäjä T., Plass-Dülmer C., Flentje H., Birmili W., Wiedensohler A., Hõrrak U. (2010). On the roles of sulphuric acid and low-volatility organic vapours in the initial steps of atmospheric new particle formation. Atmos. Chem. Phys..

