# Supplementary material for: Modeling of the Concentrations of Ultrafine Particles in the Plumes of Ships in the Vicinity of Major Harbors
Source: Int J Environ Res Public Health. 2020 Jan 26;17(3):777. doi: 10.3390/ijerph17030777 (PMC7037959; doi:10.3390/ijerph17030777)
Supplement: Supplementary file 1 [file ijerph-17-00777-s001.pdf]

Supplementary materials

Modeling of the concentrations of ultrafine particles in the plumes of ships in the vicinity of major harbors

Matthias Karl [1,*], Liisa Pirjola [2,3], Ari Karppinen [4], Jukka-Pekka Jalkanen [4], Martin Otto Paul Ramacher [1], and Jaakko Kukkonen [4]

[1] Chemistry Transport Modelling, Helmholtz Zentrum Geesthacht, 21502 Geesthacht, Germany; matthias.karl@hzg.de (M.K.), martin.ramacher@hzg.de (M.R.)

[2] Department of Technology, Metropolia University of Applied Sciences, P.O. Box 4071, FI-01600 Vantaa, Finland; liisa.pirjola@metropolia.fi (L.P.)

[3] Department of Physics, University of Helsinki, P.O. Box 64, 00014 Helsinki, Finland; liisa.pirjola@helsinki.fi

[4] Atmospheric Composition Research, Finnish Meteorological Institute, P.O. Box 503, 00101 Helsinki, Finland; ari.karppinen@fmi.fi (A.K.), jukka-pekka.jalkanen@fmi.fi (J.-P. J.), jaakko.kukkonen@fmi.fi (J.K.)

* Correspondence: matthias.karl@hzg.de

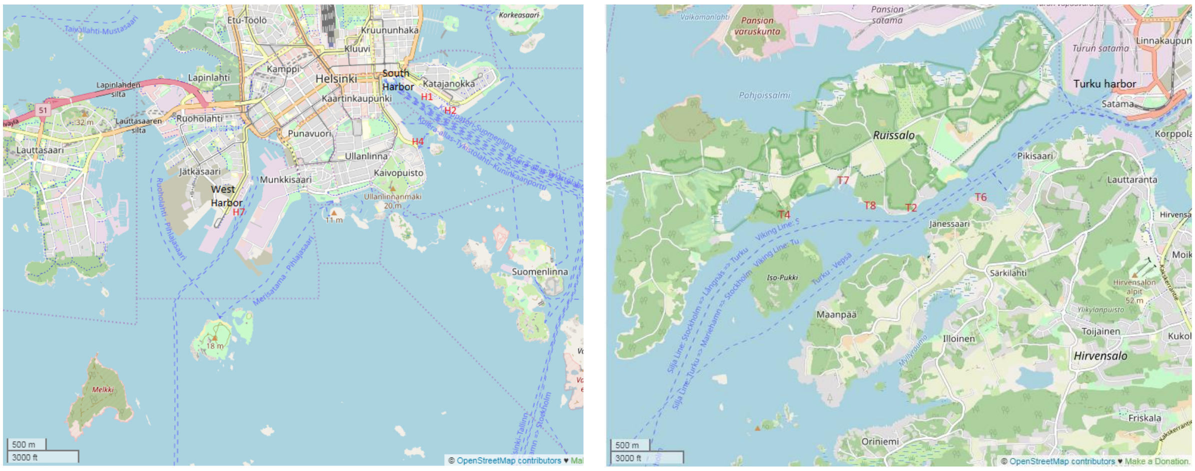

(a) (b)

Figure S1. Measurement sites in (**a**) Helsinki South Harbor (sites H1, H2 and H4) and in West Harbor (site H7), and (**b**) along the shipping channel to Turku Harbor (sites T2, T4, T6, T7 and T8). The sites are numbered as in Pirjola et al. (2014) [11]. (©OpenStreetMap contributors, CC BY-SA, see http://www.openstreetmap.org/).

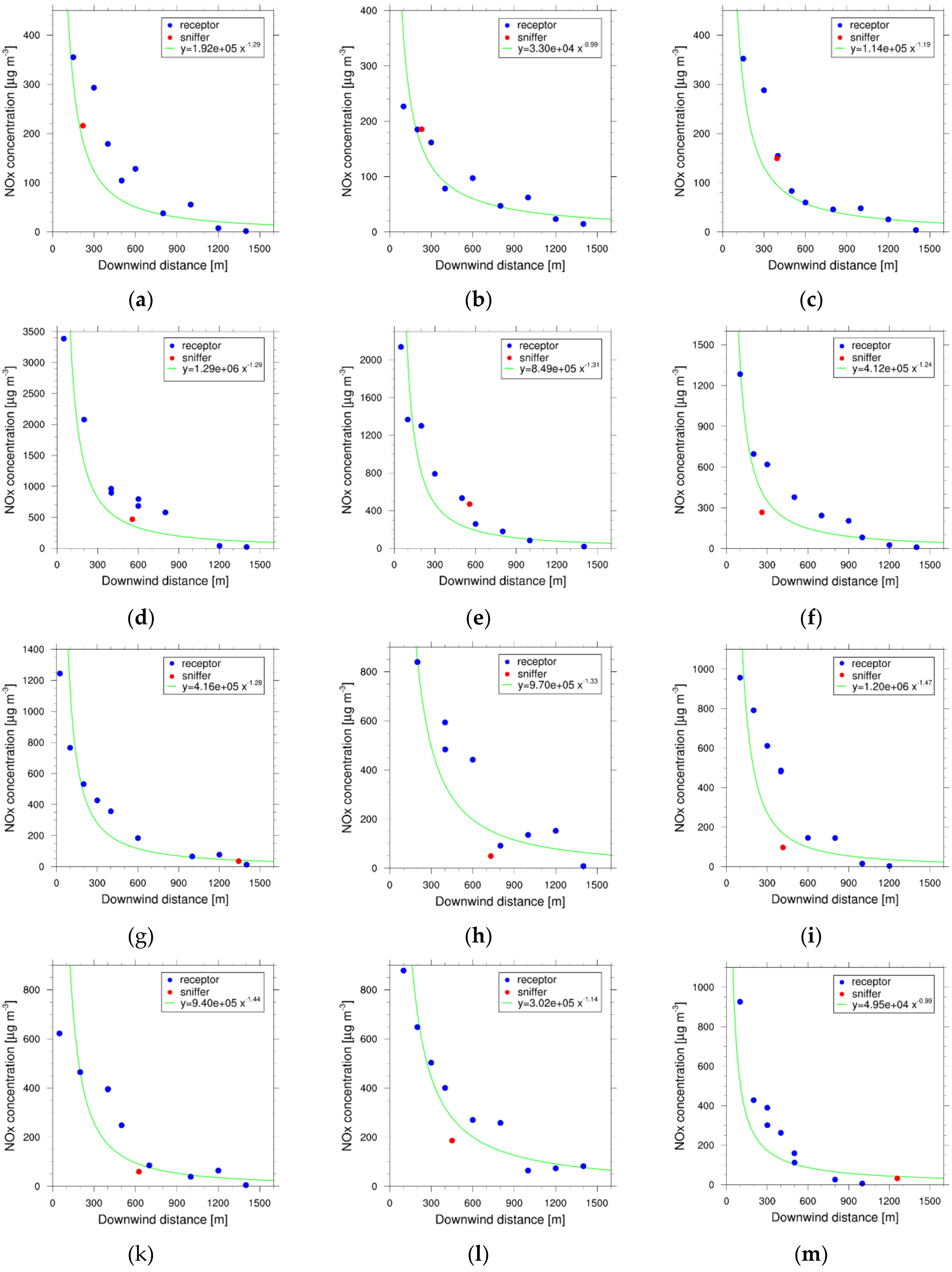

Figure S2. Power-law fit to the modeled concentrations at receptors in the ship plume based on dispersion simulations with EPISODE-CityChem: (**a**) A_20110111, (**b**) A_20110912, (**c**) A_20110913, (**d**) B_20100803, (**e**) D_20110111, (**f**) D_20110908, (**g**) H_20100811, (**h**) H_20100817, (**i**) I_20100812, (**k**) J_20100817, (**l**) J_20110217, (**m**) K_20110802. In addition, the measured peak 1-min averaged concentration at Sniffer (red dot) is indicated. Background concentrations were subtracted from the data.

(**a**)

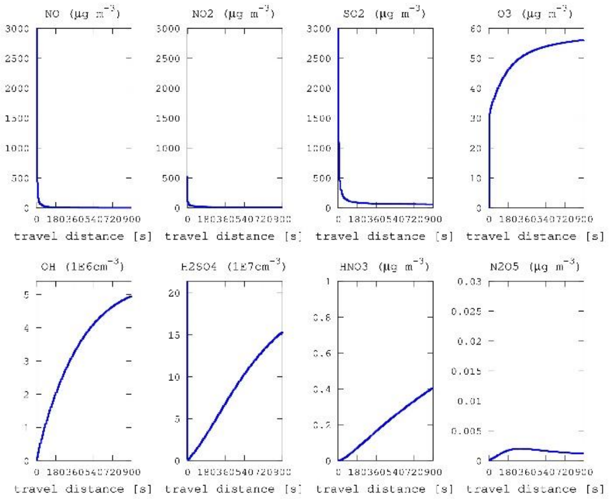

(**b**)

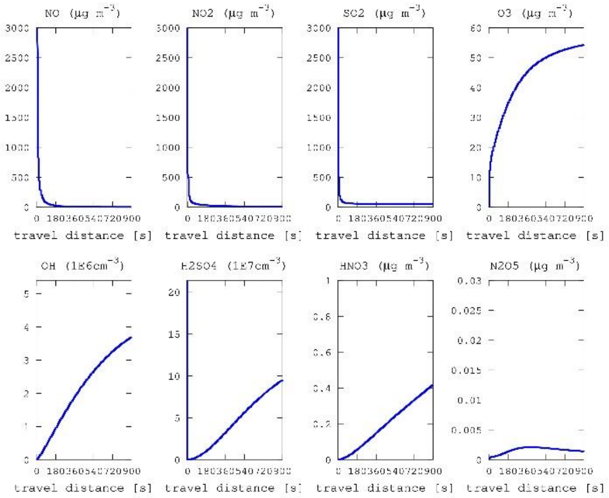

(**c**)

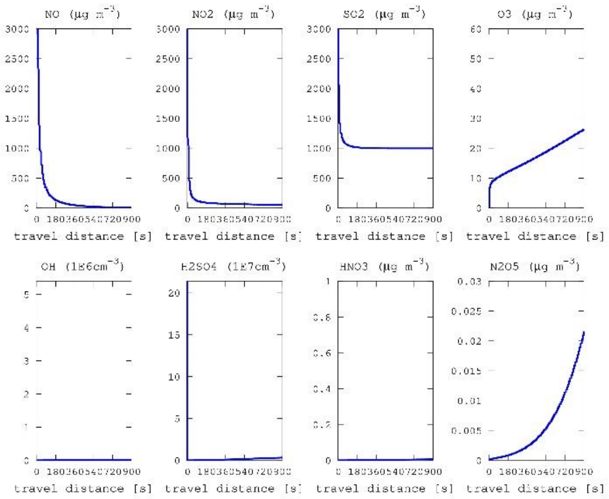

Figure S3. Gas-phase concentrations of relevant tracers and atmospheric oxidants in the ship plume in the first 15 minutes after release: (**a**) Helsinki ship event A_20110912 in summer, (**b**) Turku ship event J_20100811 in summer, and (**c**) Turku ship event J_20110217 in winter. Nitric acid (HNO_3) forming via reaction between NO_2 and OH is the main oxidation product of primary emitted NO_2 in the daytime plume, while nitrate (NO_3) radicals lead to increasing production of dinitrogen pentoxide (N_2O_5) in the nighttime plume.

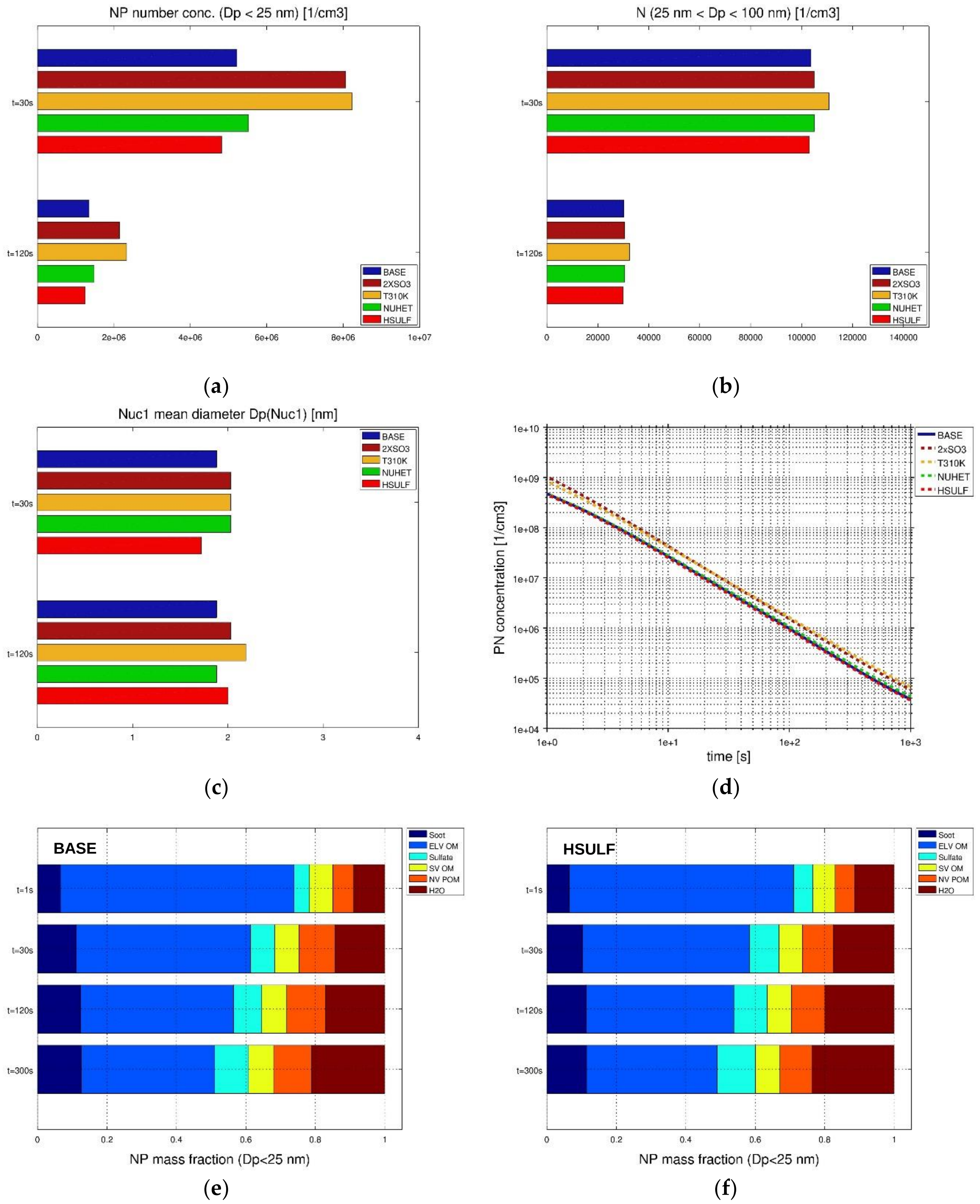

Figure S4. Results from the sensitivity tests with the aerosol dynamics model. (**a**) NP number concentration after 30 s and 120 s plume travel time, (**b**) number concentration of ultrafine particles with Dp > 25 nm (after 30 s and 120 s), (**c**) mean diameter of Nuc_1 mode (after 30 s and 120 s), (**d**) PN concentration as function of time on logarithmic scale, (e) chemical composition of nanoparticles (wet particle mass) in the base run after 1 s, 30 s, 120 s, 300 s ; and (**f**) the same for case HSULF. Sensitivity tests were performed for ship event A_20110912. BASE: base run configuration; 2XSO3: SO_X-to-SO_3 conversion rate of 2%; T310K: initial in-plume temperature of 310 K; NUHET: homogeneous heteromolecular nucleation between H_2SO_4 and organic vapor molecules; HSULF: Nuc_2 mode mass composed of 100 % liquid H_2SO_4.

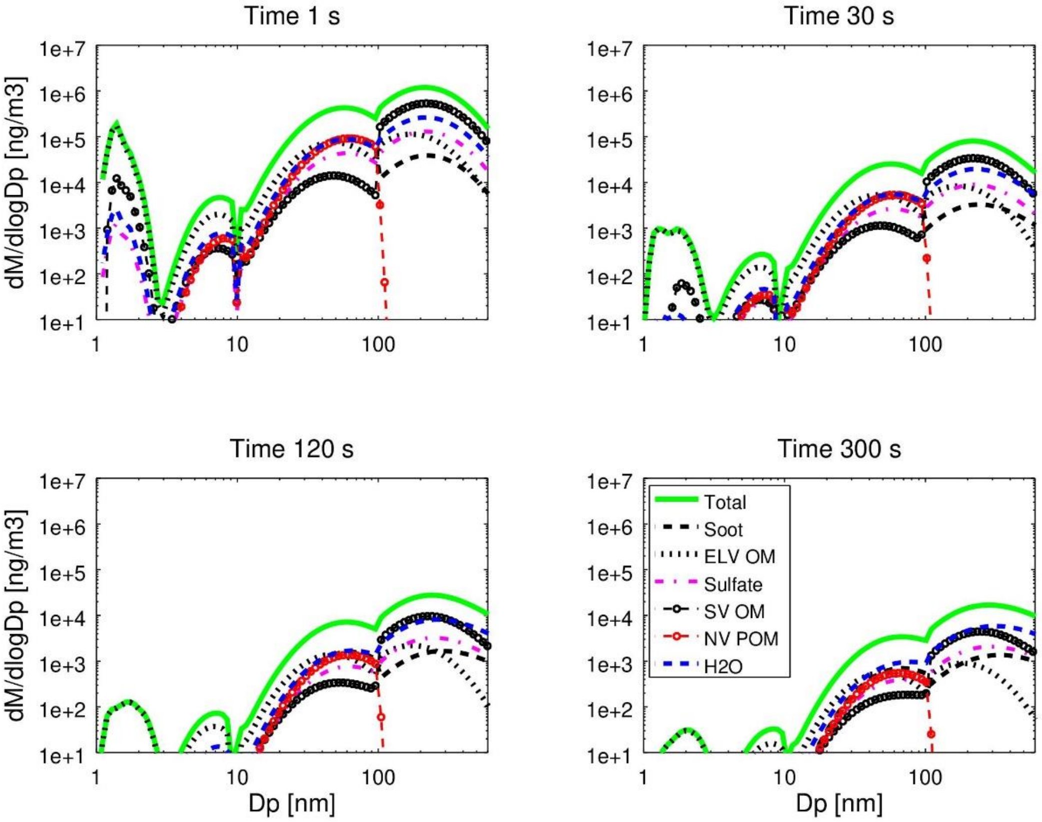

Figure S5. Mass composition distribution in the size range 1−600 nm at different travel times of the ship plume simulated with the MAFOR model. In addition to the total mass concentration (green line), the plots show the mass distributions of nonvolatile primary organic matter (NV-POM; red dashed line with open circles), soot (black dashed line), sulfuric acid (magenta dash dotted line), semivolatile and low volatile organic matter (SV-OM, black dashed line with open circles), extremely low-volatility organic matter (ELV-OM) as well as the mass distribution of water (blue dashed line). Results are from the base simulation for ship event A_20110912.

Table S1. Ship stack parameters and ship geometry. Stack height (H_S), stack diameter (D_S), exhaust temperature (T_E), exhaust exit velocity (V_S), ship building height (H_B), ship building width (W_B).

Ship name	H_S (m)	D_S [1] (m)	T_E (K)	V_S (m s^{-1})	H_B (m)	W_B (m)
**A** (Viking Express)	33	0.8	580	23	28 [2]	28
**B** (Baltic Princess)	45	0.8	580	23	40 [2]	29
**D** (Viking Mariella)	45	0.8	580	23	40 [2]	28
**H** (Silja Galaxy)	45	0.8	580	23	40 [2]	29
**I** (Viking Isabella)	45	0.8	580	23	40 [2]	28
**J** (Viking Amorella)	45	0.8	580	23	45	28
**K** (Seawind)	40	0.8	580	23	35 [2]	22

[1] A circular stack exit is assumed.

[2] Building height estimated as being 5 m below stack exit.

Table S2. Ship engine power (ME: main engine, AE: auxiliary engine) and ship emissions of NO_X, SO_X and total particle number (PN) based on STEAM during ship events. Values are 1-min. averages during ship passage, when the ship was closest to Sniffer.

Event	ME (kW)	AE (kW)	E(NO_X) (g s^{-1})	E(SO_X) (g s^{-1})	E(PN) (s^{-1})
A_20110111	4100	1460	1.4	2.9	$0.72x10^{15}$
A_20110912	900	1460	0.5	1.0	$0.50x10^{15}$
A_20110913	3050	1460	1.0	2.2	$1.07x10^{15}$
B_20100803	0	3530	11.8	0.4	$1.05x10^{15}$
D_20110111	0	3270	10.9	0.4	$0.99x10^{15}$
D_20110908	10	3270	10.9	0.4	$0.97x10^{15}$
H_20100811	1570	3530	12.3	2.3	$1.50x10^{15}$
H_20100817	1770	3530	12.4	2.5	$1.56x10^{15}$
I_20100812	1060	2440	11.9	1.6	$1.05x10^{15}$
I_20110207	1080	2440	12.0	2.2	$1.06x10^{15}$
J_20100811	1250	2440	12.6	1.9	$1.11x10^{15}$
J_20100817	940	2440	11.6	1.5	$1.03x10^{15}$
J_20110217	970	2440	12.7	2.1	$1.12x10^{15}$
K_20110802	690	700	4.8	0.9	$0.44x10^{15}$

Table S3. Mass composition (in %) of the ship exhaust particle emissions. Size ranges of the particle modes: Nuc_2 – Dp < 10 nm; Aitken – 10 nm < Dp < 100 nm; Acc – 100 nm < Dp < 1000 nm; Coarse – 1000 nm < Dp < 8000 nm.

Particle size mode	Sulfuric acid	Organic carbon	Elemental carbon	Mineral dust	Sea salt	Ammonium nitrate
**Nuc2**	20	80	0	0	0	0
**Aitken**	16	40	44	0	0	0
**Acc**	16	67	5	12	0	0
**Coarse**	16	65	5	14	0	0

Table S4. Mass composition (in %) of particulate matter in the urban background air. Size ranges of the particle modes: Nuc_2 – Dp < 10 nm; Aitken – 10 nm < Dp < 100 nm; Acc – 100 nm < Dp < 1000 nm; Coarse – 1000 nm < Dp < 8000 nm.

Particle size mode	Sulfuric acid	Organic carbon	Elemental carbon	Mineral dust	Sea salt	Ammonium nitrate
**Nuc2**	26	68	0	0	0	6
**Aitken**	23	18	20	12	5	22
**Acc**	23	18	20	12	5	22
**Coarse**	23	18	20	12	5	22

Table S5. Conditions of the ship plume dispersion and parameters used in the single term power series fits to the modeled NO_X concentration data for ship events in Helsinki and Turku. The atmospheric stability conditions (according to the P-G classification) was determined based on the inverse Monin-Obukhov length (L_O^{-1}) calculated by MPI-FMM; H_{mix} is the mixing height and H_{eff} is the effective emission height.

Event	Atmospheric stability, P-G	L_O^{-1} (m^{-1})	H_{mix} (m)	H_{eff} (m)	a	b
A_20110111	neutral, D	4.95×10^{-4}	638	14	0.19×10^{6}	1.29
A_20110912	slightly unstable, C	-8.01×10^{-5}	519	14	0.03×10^{6}	0.99
A_20110913	slightly unstable, C	-2.36×10^{-4}	2020	14	0.11×10^{6}	1.19
B_20100803	unstable, B	-1.04×10^{-2}	1140	48	1.29×10^{6}	1.29
D_20110111	neutral, D	5.45×10^{-4}	661	32	0.85×10^{6}	1.31
D_20110908	very stable, F	4.59×10^{-2}	131	87	0.41×10^{6}	1.24
H_20100811	unstable, B	-1.19×10^{-2}	1060	41	0.42×10^{6}	1.28
H_20100817	unstable, B	-6.80×10^{-3}	674	42	0.97×10^{6}	1.33
I_20100812	unstable, B	-2.95×10^{-2}	1620	84	1.20×10^{6}	1.47
I_20110207	slightly stable, E	3.10×10^{-3}	194	43	1.19×10^{6}	1.44
J_20100811	unstable, B	-1.19×10^{-2}	1060	38	0.59×10^{6}	1.27
J_20100817	unstable, B	-6.80×10^{-3}	674	44	0.94×10^{6}	1.44
J_20110217	very stable, F	3.50×10^{-1}	64	44	0.30×10^{6}	1.14
K_20110802	very stable, F	1.14×10^{-1}	95	84	0.05×10^{6}	0.99

Table S6. Comparison of modeled and measured PN concentrations at peak time t1, for ship events in Helsinki and Turku. PN includes all particles with Dp > 10 nm in size. Travel time dt is the time an air parcel released at ship stack needs to reach Sniffer; dt = t1 - t0. Distance of the ship to Sniffer at t1 is based on the AIS position data.

Event	Peak time t_1	Travel time dt (s)	Distance ship (m)	Observed PN (cm^{-3})	Modeled PN (cm^{-3})	M-O (%)
A_20110111	11:37:33	22	220	$11.8x10^4$	$16.2x10^4$	37
A_20110912	11:40:47	42	231	$27.0x10^4$	$16.5x10^4$	-39
A_20110913	11:33:43	79	394	$29.5x10^4$	$7.62x10^4$	-74
B_20100803	18:36:40	103	556	$4.25x10^4$	$3.63x10^4$	-15
D_20110111	17:44:45	69	348	$7.07x10^4$	$4.43x10^4$	-37
D_20110908	17:42:28	130	261	$6.34x10^4$	$8.97x10^4$	41
H_20100811	08:43:20	240	1342	$2.36x10^4$	$2.24x10^4$	-5
H_20100817	08:35:30	193	732	$2.43x10^4$	$3.25x10^4$	34
I_20100812	19:31:40	139	416	$4.48x10^4$	$2.67x10^4$	-40
I_20110207	19:26:50	335	1273	$2.11x10^4$	$0.61x10^4$	-71
J_20100811	09:05:00	269	1143	$2.77x10^4$	$2.22x10^4$	-20
J_20100817	09:01:50	93	625	$1.86x10^4$	$2.72x10^4$	46
J_20110217	07:14:50	196	451	$8.24x10^4$	$8.32x10^4$	1
K_20110802	20:23:50	1797	1258	$0.79x10^4$	$1.20x10^4$	51
